# African dust transport and deposition modelling verified through a citizen science campaign in Finland

**DOI:** 10.1038/s41598-023-46321-7

**Published:** 2023-12-04

**Authors:** Outi Meinander, Rostislav Kouznetsov, Andreas Uppstu, Mikhail Sofiev, Anu Kaakinen, Johanna Salminen, Laura Rontu, André Welti, Diana Francis, Ana A. Piedehierro, Pasi Heikkilä, Enna Heikkinen, Ari Laaksonen

**Affiliations:** 1 Finnish Meteorological Institute, Climate Research, Erik Palmenin Aukio 1, 00560 Helsinki, Finland; 2 Department of Geosciences and Geography, University of Helsinki, Gustaf Hällströmin Katu 2, 00560 Helsinki, Finland; 3 Geological Survey of Finland (GTK), Vuorimiehentie 2, 02150 Espoo, Finland; 4 Finnish Meteorological Institute, Meteorological Research, Erik Palmenin Aukio 1, 00560 Helsinki, Finland; 5 Finnish Meteorological Institute, Research Coordination, Erik Palmenin Aukio 1, 00560 Helsinki, Finland; 6 Earth Sciences Department, Khalifa University, 127788 Abu Dhabi, United Arab Emirates; 7 Department of Technical Physics, University of Eastern Finland, 70210 Kuopio, Finland

**Keywords:** Chemical physics, Atmospheric chemistry, Environmental monitoring

## Abstract

African desert dust is emitted and long-range transported with multiple effects on climate, air quality, cryosphere, and ecosystems. On 21–23 February 2021, dust from a sand and dust storm in northern Africa was transported to Finland, north of 60°N. The episode was predicted 5 days in advance by the global operational SILAM forecast, and its key features were confirmed and detailed by a retrospective analysis. The scavenging of dust by snowfall and freezing rain in Finland resulted in a rare case of substantial mineral dust contamination of snow surfaces over a large area in the southern part of the country. A citizen science campaign was set up to collect contaminated snow samples prepared according to the scientists’ instructions. The campaign gained wide national interest in television, radio, newspapers and social media, and dust samples were received from 525 locations in Finland, up to 64.3°N. The samples were utilised in investigating the ability of an atmospheric dispersion model to simulate the dust episode. The analysis confirmed that dust came from a wide Sahara and Sahel area from 5000 km away. Our results reveal the features of this rare event and demonstrate how deposition samples can be used to evaluate the skills and limitations of current atmospheric models in simulating transport of African dust towards northern Europe.

## Introduction

Wind-blown dust particles derived from land surfaces can play an important role in atmospheric processes and in modifying Earth’s climate^1^. Dust significantly influences the radiation balance of the atmosphere^2^ and is important and effective in the nucleation of ice crystals^3,4^. Dust can further provide nutrients, such as iron, nitrogen, and phosphorus to both marine and terrestrial ecosystems^5,6^. When dust is transported and deposited on snow and ice^7^, the ensuing darkening of surfaces lowering of albedo can result in radiative forcing over time periods lasting from minutes to centuries^8–10^. North Africa is the largest and most significant terrestrial source of dust, with a multitude of impacts on solar energy production, agriculture, ground and air transportation, society, health and emergency response systems^11^. This region forms part of the so-called “global dust belt” (GDB)^12^, defined as extending into the Northern Hemisphere from the western coast of northern Africa over the Middle East (western Asia), central, and East Asia and southwestern North America^13^; sources in the Southern Hemisphere are relatively minor by comparison^12,13^.

Finland is also affected by long-range transport of African dust. Most often this dust is observed as red sunrises and sunsets, while observations of deposition of dust on the ground are extremely rare. The current study considers a recent event of the Saharan dust transport towards Finland in February 2021.

There is only one previously documented case of Saharan dust deposition in Finland, from the northern part of the country, above the Arctic circle at 66.5°N, on 8–10 March 1991^14^. They estimated that during this event, northernmost Europe was affected over an area of at least 320,000 km^2^ with a total of 50,000 tonnes of dust deposited. Accordingly, the March 1991 sand and dust storm (SDS) event was one of the largest Saharan dust events recorded in the 1900s, affecting central Europe and northern Scandinavia, only surpassed, or equalled by, the “blood rain” of 1901 when 1–4 g/m^2^ Saharan dust was deposited as far north as northern Germany. At that time, warm winds were blowing from the Sahara for a period of several days; the airflow was very strong and turbulent, forced by a high-pressure gradient between an anticyclone over north-eastern Africa and a strong depression moving in over the Atlas region^14^. On 10 March 1991, a brownish-yellow colour was observed on the snow cover in northern Scandinavia, around the northern part of the Gulf of Bothnia in Finland and Sweden. This caused widespread concern over contamination and pollution amongst local inhabitants and many members of the public collected and sent dust and snow samples to the health authorities for analysis. The event was also reported on radio and in newspapers in Finland and Sweden, indicating the importance of citizen awareness and engagement. Analysis of the samples submitted by the public revealed the presence of mineral particles, loose ferric aggregates of mineral grains and abundant pollen and spores; particle sizes ranged between 1 and 7 µm with a median of 2.7 µm, with a few aggregates attaining a diameter of 10 µm. The composition of the organic compounds in the yellow dust indicated that they had been adsorbed by the particles during transportation^14^.

During the northern winter of February 2021, an extreme atmospheric event transported dust from Northern Africa towards Europe. Two successive major Saharan dust outbreaks were reported for 4–8 February and 18–25 February, affecting large areas of western Europe, turning both the sky and snow cover orange, extending all the way to Scandinavia, notably Denmark and southern Sweden^10^. They identified a new pathway of atmospheric rivers (ARs) towards Europe: across the Sahara Desert, with the ARs originating in the eastern Atlantic Ocean. Atmospheric rivers refer to the filamentary structure of atmospheric water vapor transport^15^, which advect low-latitude air and moisture poleward^16,17^. According to^10^, during the last four decades, 78% of the atmospheric rivers occurring over northwest Africa were associated with extreme dust events over Europe.

On 21–23 February 2021, African dust was transported and deposited over extensive areas in southern Finland, north of 60°N, in coastal districts and penetrating a considerable distance inland^18^. The 21–23 February event was forecasted five days in advance by the Finnish Meteorological Institute’s (FMI) operational SILAM (System for Integrated modeLling of Atmospheric coMposition) global atmospheric-composition suite^19^. At the time, the country was under an extensive blanket of snow, and therefore the accumulation of dust was more easily discernible. The Finnish Meteorological Institute initiated a citizen science campaign because of several public enquiries asking for explanations as to why the snow was coloured. The widespread occurrence of dust and the citizen science Saharan dust sampling campaign received wide national attention and interest at television, radio, newspapers, and social media.

For citizen science campaigns in Finland, winter season dust events offer great opportunities for collecting dust samples to support investigations into modelled and satellite-based observations of long-range transport and deposition processes, and to quantify the radiative forcing effects of dust on snow and ice. Below, we describe the meteorological circumstances that led to African dust transport to Finland and the analysis of sources, transport and deposition of dust using SILAM dispersion modelling. We compare the model results with the observations from the citizen science campaign and discuss the results of the comparison in the light of observed particle properties, such as, magnetic properties, mineral composition, and particle size distributions. The analysed samples were selected to represent different geographical locations and sampling methods (Fig. 7 and Supplementary Table 1).

## Results

### Meteorological situation leading to the transport event

**Figure 1:**
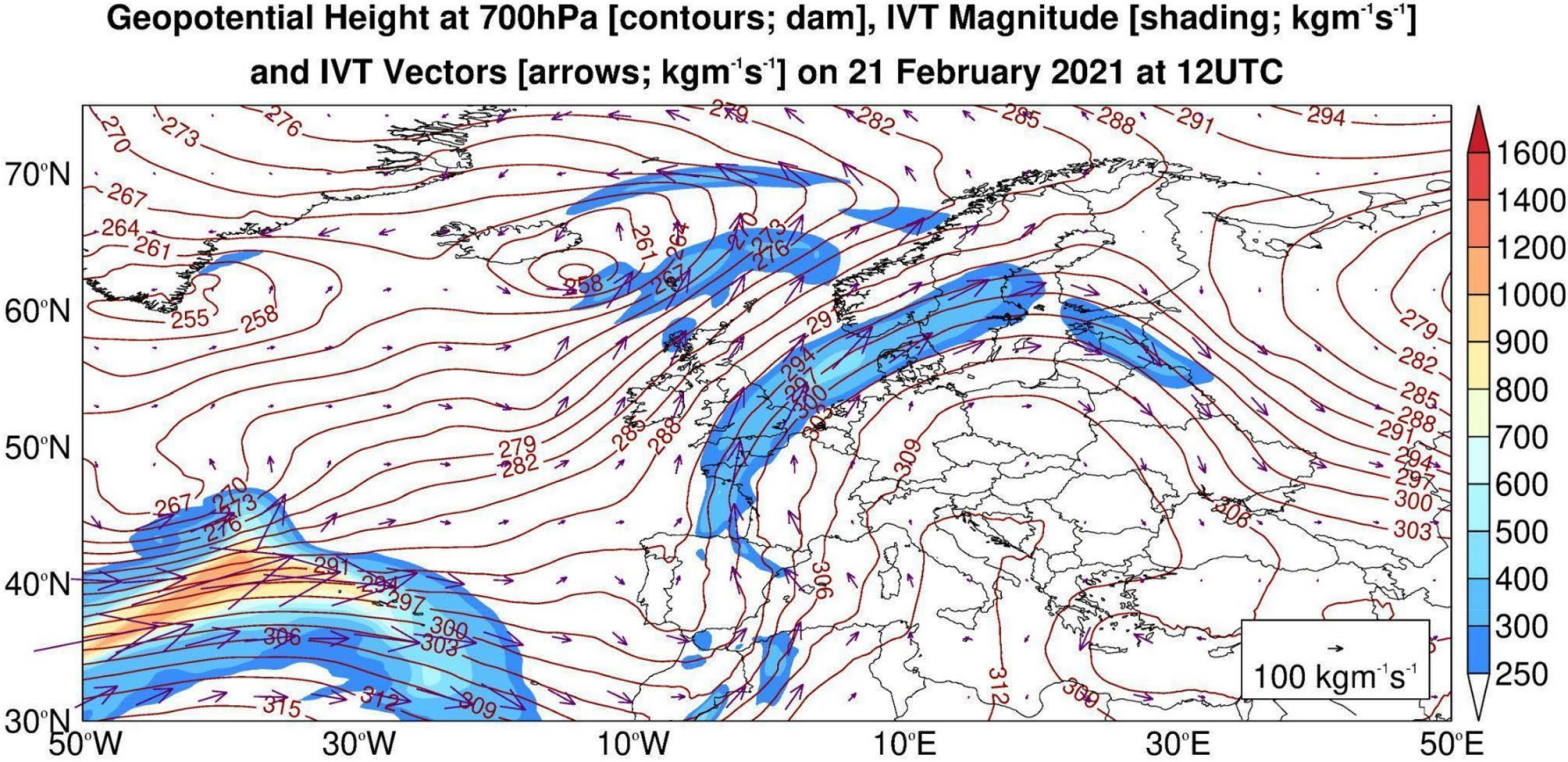
ERA5 geopotential height at 700hPa and integrated water vapor transport (IVT) on 21 February 2021 at 12 UTC. The map was created using IDL. Version: 8.8.0. URL Link: https://www.nv5geospatialsoftware.com/Products/IDL/.

The synoptic situation on 21 to 23 February 2021 was characterised by a strong anticyclone over Central Europe and a low-pressure trough over the Eastern Atlantic. The ARs described in^10^ were still visible over Finland (Fig. 1).

**Figure 2:**
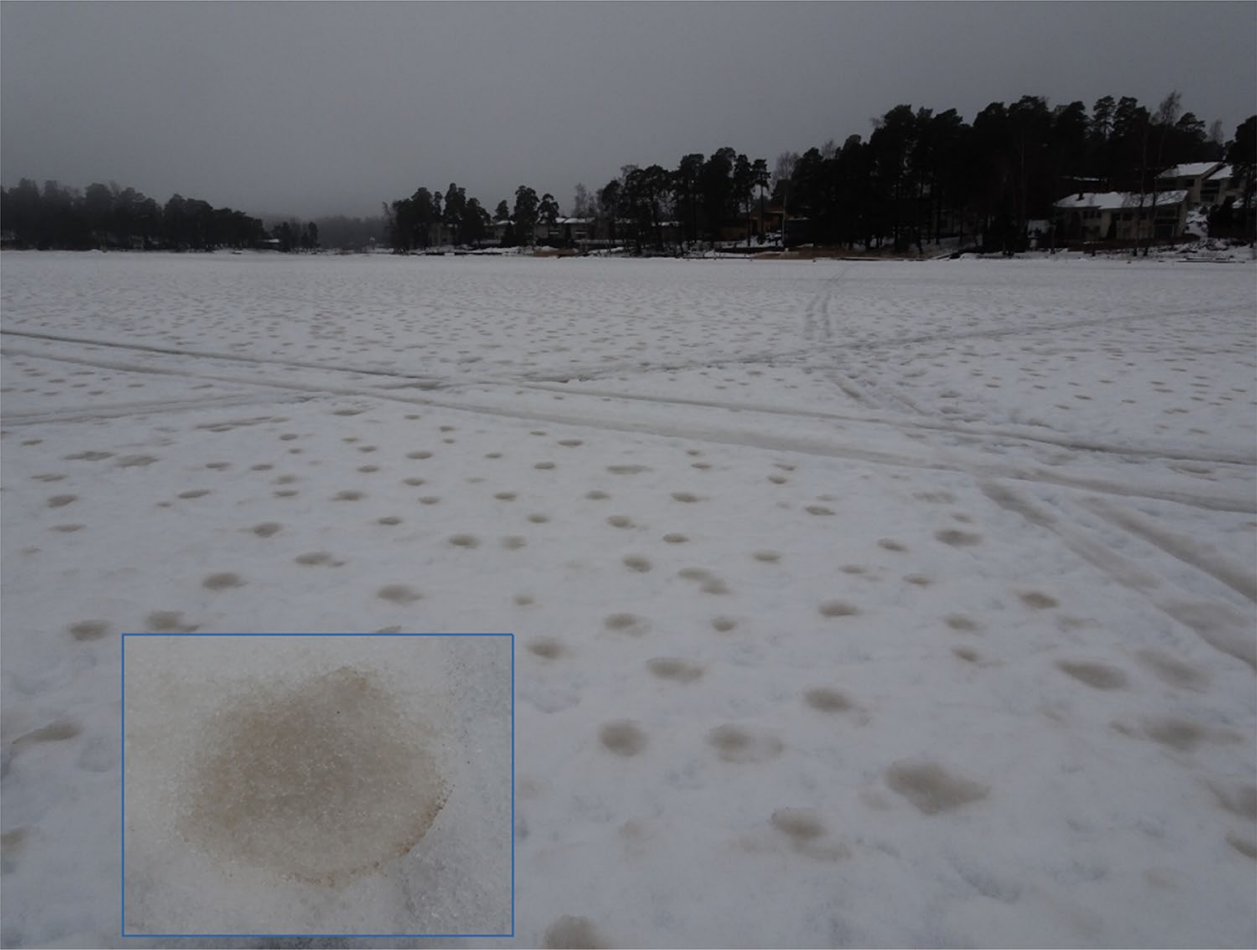
Saharan dust residue after thawing of snow on sea ice at Helsinki (60.17° N, 24.94° E) on 25 February 2021. Diameter of the dust-stained patches was around 5 cm.

The ARs passing over the Sahara triggered strong near-surface winds and favoured entrainment and rapid northward transport of dust towards Scandinavia between the Pyrenean and Alpine mountains. During 22 February, a smaller low-pressure system with frontal precipitation developed over the Baltic Sea and moved over Finland from the southwest. Intense warm advection prevailed during the following days, which led to the melting of all snow on the frozen Baltic Sea ice offshore from Helsinki, Finland. Residual patches of Saharan dust remained on the melting snow (Fig. 2).

### Meteorological situation during the dust event

**Figure 3:**
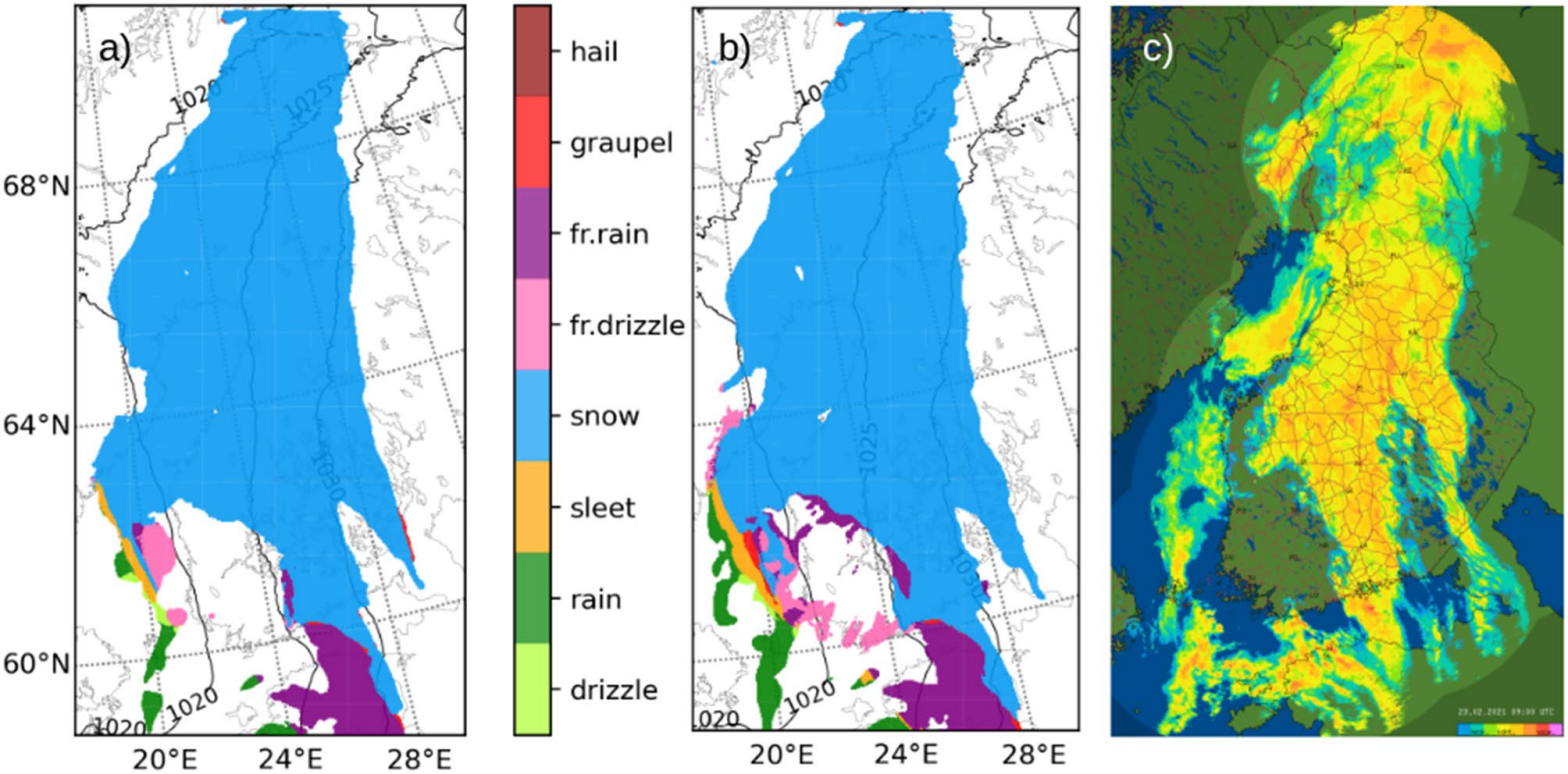
HARMONIE-AROME Numerical Weather Prediction model precipitation type output for 09 UTC on 23 February 2021, three-hour forecast based on 06 UTC initial time: (**a**) default experiment, (**b**) experiment with CAMS aerosol. (**c**) Precipitation intensity from FMI weather radar composite valid at 09 UTC. The prevailing yellow tones indicate moderate intensity. The maps were created using FMI operational radar software.

The operational high-resolution (horizontal resolution 2.5 km) numerical weather forecast of the FMI is run within the framework of Nordic MetCoOp cooperation^20^, based on the HARMONIE-AROME NWP model^21^. The forecasts valid at 09 UTC (11 CET) on 23 February 2021 suggested precipitation over whole Finland, with a minimum in southwest (Fig. 3a). The distribution of precipitation corresponded well to the FMI weather radar observations (Fig. 3c). Measured accumulated precipitation at weather stations between 18 UTC on 22 and 18 UTC on 23 February over Finland varied from minimum values of 0.1 mm (kg/m^2^ water equivalent) in the southwest to maximum values of 9.6 mm in southeast of Finland. Corresponding values from the model were 1 and 15 mm. The forecast showed an interesting interpretation of precipitation type during the dust intrusion. Rain, freezing rain and drizzle, sleet, graupel and snow were all suggested over various parts of the forecast domain (Fig. 3a). According to the weather observations, all these precipitation modes were in fact observed during the day, especially over coastal areas, on the Baltic Sea and sea ice. The operational weather model does not simulate aerosol impact on cloud-radiation-precipitation processes. Near-real-time aerosol data from Copernicus Atmosphere Monitoring Service (CAMS) were included and tested in a development version of HARMONIE-AROME (model version CY46h1). Figure 3 shows an example of precipitation type in two experiments valid at 09 UTC (11 CET) on 23 February: default without aerosols influencing clouds (Fig. 3a) and an experiment that included CAMS near-real-time mass mixing ratio data on 14 aerosol species (Fig. 3b). For comparison, the precipitation intensity observed by FMI weather radars is shown (Fig. 3c). When only dust aerosol was included, the amount of liquid precipitation (rain) increased at the expense of solid precipitation (snow, graupel) in southwestern Finland while the total precipitation was only little affected. The effect is related to the way dust is treated by the cloud microphysics parametrizations of the model and requires further evaluation.

### Dispersion modelling

**Figure 4:**
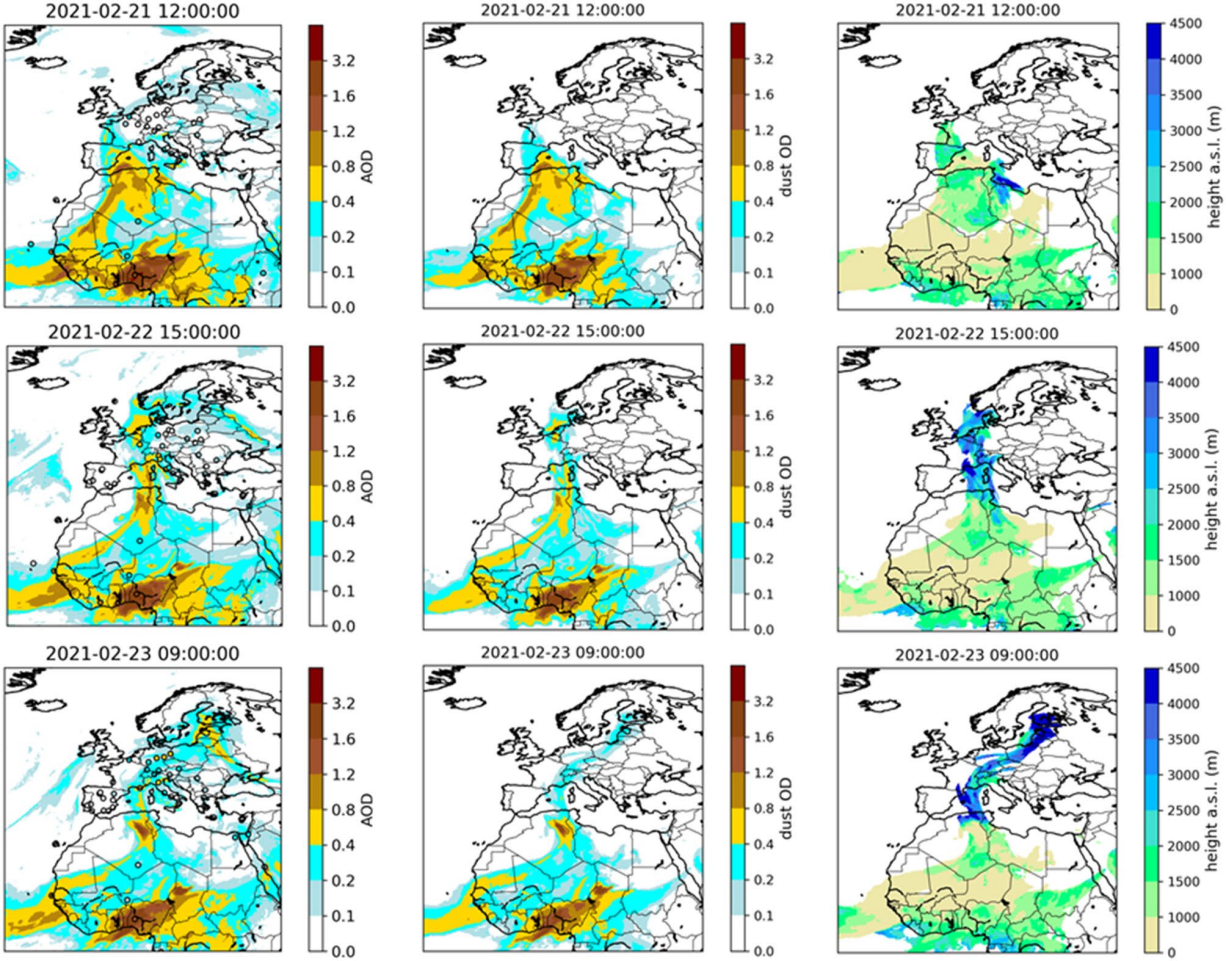
Total AOD at 550 nm (left column), dust optical depth at 550 nm (centre column) and height of centre of mass of the main mode of dust (right column) simulated using the SILAM model for three separate times. Areas with dust optical thicknesses of less than 0.1 have been cut out from the height plots. The total AOD is evaluated against the AOD measured at AERONET stations located at the circular markers, with the marker face colour indicating the AERONET measured value. The maps were created using Python 3.10.12, python3-cartopy 0.20.2.

The transport of dust to Finland by this event was forecasted five days in advance by the operational SILAM global atmospheric-composition suite of the FMI, driven by the ECMWF IFS operational meteorological forecast. The development of the event, based on the SILAM model results, is illustrated in Fig. 4, together with aerosol optical depth (AOD) measurements from the AERONET network. On February 21, the AR that had formed north of Algeria dragged a plume of dust northward at a height (defined as the height of the centre of mass of the main dust modes) of roughly 2000 m. On the following day, the plume had reached the North Sea while simultaneously gaining altitude. On the morning of February 23, its northern tip was located above southern Finland at an average height that exceeded 4000 m (described in more detail by Supplementary Fig. S2). Based on a dispersion forecast by SILAM, over northern Europe, the dust plume was already significantly contaminated with other aerosols, which is consistent with the retrieval of the aerosol subtype by the lidar carried by the CALIPSO satellite, shown in Supplementary Fig. S3. The CALIPSO derived plume height over central Europe is also consistent with the SILAM results, while the clouds blocked the lidar above the Baltic Sea and Finland.

The model results show that the near-surface concentrations of desert dust in Finland were negligible, while the dust optical depth reached values exceeding 0.2 in the southern part of the country, with the total AOD exceeding 0.4, which is well enough to be visible. The scavenging of dust from higher levels in the atmosphere resulted in substantial contamination of the precipitating snow.

**Figure 5:**
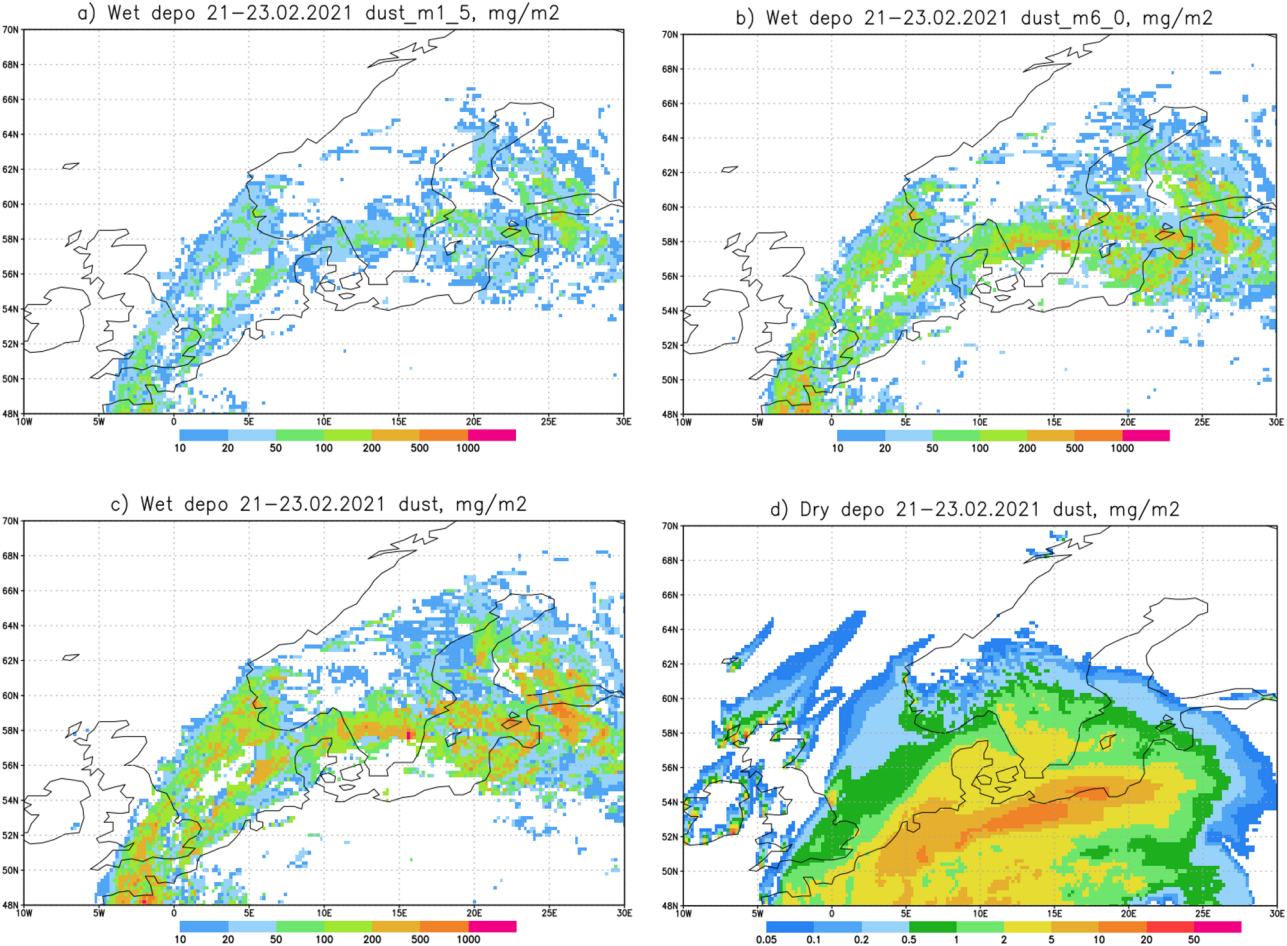
The results of SILAM modelling of dust deposition [mg/m^2^] over northern Europe and Fennoscandia on 21–23 February 2021: (**a**) wet deposition of particles in the 1.5 µm size bin, (**b**) wet deposition of particles in the 6 µm size bin (**c**) total wet deposition, and (**d**) dry deposition. Note the different colour scales in (**a**–**c**) and (**d**). The maps were created using Grid Analysis and Display System (GrADS) Version 2.2.1.

The wet and dry deposition of the Saharan dust event were also modelled by SILAM through the estimation of deposition amounts [mg/m^2^] for different particle size ranges (Fig. 5). The results showed wet deposition of dust over a large area in the southern part of Finland, and either minimal, or a complete lack of dry deposition over Finland. The maximum total wet deposition of dust on February 21–23 was 500–1000 mg/m^2^ at several locations, including, e.g., the southern coast and southernmost inland areas. This was accompanied by additional areas of dust accumulation outside Finland, such as on Saarenmaa, an Estonian island in the Baltic Sea 130 km southwest of Helsinki, for which a maximum dust deposition of 500–1000 mg/m^2^ was modelled (Fig. 5a). Particles of the size bin centred at 6 µm, covering the size range of 2.5–10 µm, were found to form the prevailing mode of this depositional event (Fig. 5b). The maximum deposition for particles in this size bin was 500–1000 mg/m^2^ over the southern Finland town of Tampere (61.50° N, 23.76° E), as well as over some additional locations. For particles sized 1.0–2.5 µm, the maximum deposition was 200–500 mg/m^2^ over southern inland areas, surrounded by areas with a deposition of 100–200 mg/m^2^ (Fig. 5c). Again, modelling showed no dry deposition over Finland (Fig. 5d).

In addition to deposition modelling, the SILAM total-column mass of dust aerosol [g/m^2^] was modelled for four size classes of 0.01–1 µm, 1–2.5 µm, 2.5–10 µm, 10–30 µm, and for their aggregate sum. The maximum dust load over Helsinki sea ice (60.17° N, 24.94° E) was around 0.15 g/m^2^, while it was 0.25 g/m^2^ over Tampere. Supplementary Table S4 summarises the model-based optical depth, total aerosol column and average deposition for dust and other aerosol species over southern Finland during the event.

**Figure 6:**
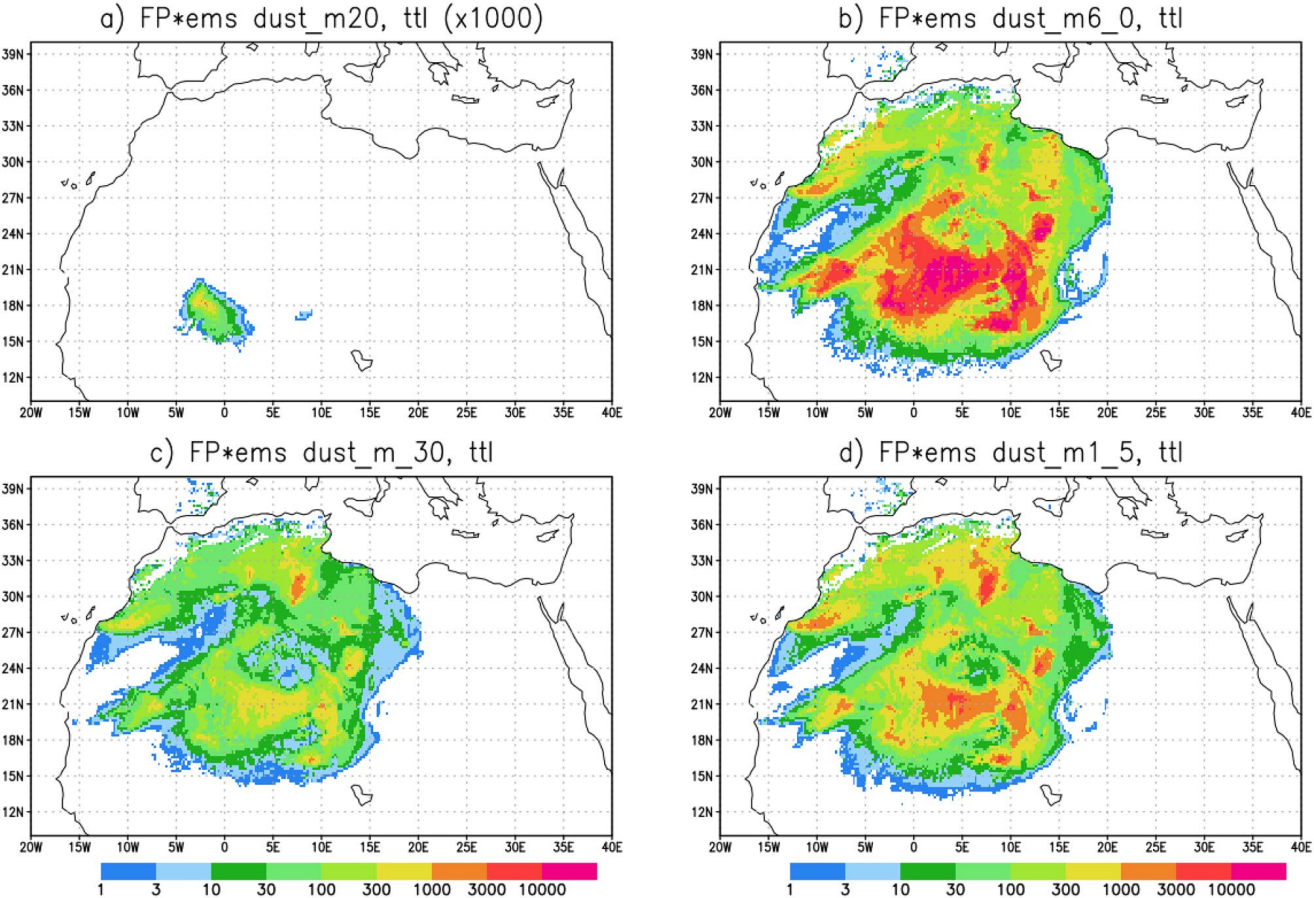
SILAM footprints convolved with the emission indicate an extensive area of northwestern Africa as the source of the dust that was deposited in Finland. The panels correspond to the four bins used in the computations (m20:10–30 µm; m6_0: 2.5–10 µm, m_30: 0.01–1.0 µm; m1_5: 1.0–2.5 µm). Note the intensity scale in (**a**) is magnified by 1000. The maps were created using Grid Analysis and Display System (GrADS) Version 2.2.1.

SILAM source inversion indicated that the dust originated from a wide area in the Sahara and Sahel and was emitted over a period of three days, i.e., February 16–18 (Fig. 6). The source estimation is based on a convolution of an adjoint model run (a footprint), calculated backwards in time for each dust size bin from the morning of February 23 and from an altitude of 4500 m above southern Finland, with the SILAM modelled dust emission. The figure also highlights that in the model, the coarsest size bin of dust did not disperse to Finland from the emission regions in Sahara but was instead deposited along the way.

Weighting the SILAM estimated sources of Fig. 6 with data from the Harmonized World Soil Database version 2.0^22^ yields that the dominant topsoil (0–20 cm below the surface) type of the source was arenosol (41%), followed by regosol (35%), leptosol (11%), calcisol (4.8%) and gypsisol (1.5%). The actual emission likely corresponds to a somewhat different distribution than this, as e.g. the leptosols cover areas with rocks and bare cliffs. The average physicochemical features for the source, given by the database, are summarised in Supplementary Table S5.

### Citizen observations

**Figure 7:**
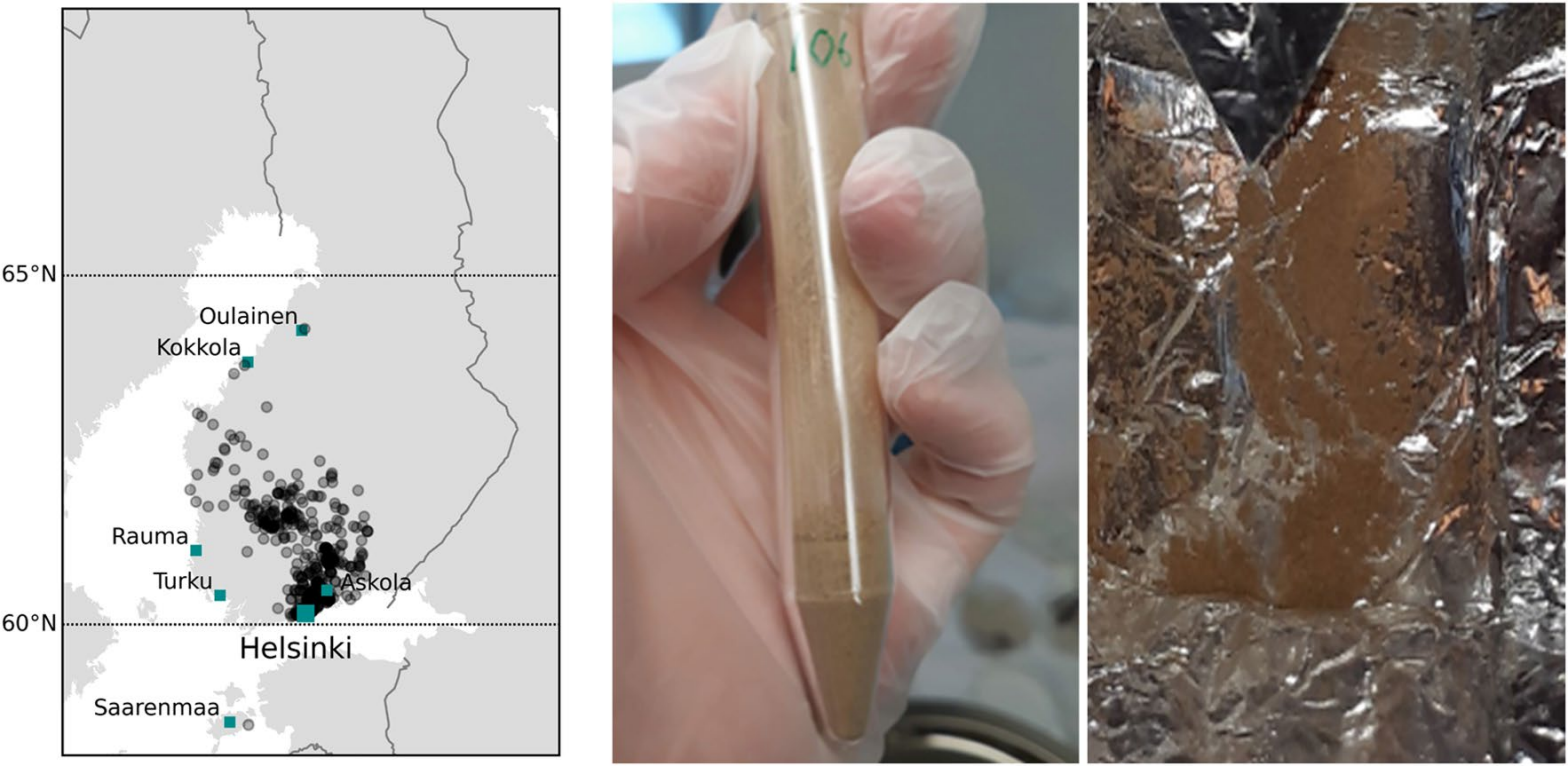
Left: Geographical distribution of the samples collected by citizens. The blue-green squares in the map indicate locations mentioned in the body text. Middle: Evaporated dust sample 106 from Askola, 54 km from Helsinki and 20 km north from the south coast. Right: Evaporated dust sample from Saarenmaa, an Estonian island 130 km south from Helsinki. The map was made in Python 3.9.7, using basemap 1.3.6, https://matplotlib.org/basemap/index.html.

Citizen samples of dust were received from 525 locations throughout southern and western Finland (Fig. 7) and each of these contained one or more filtered, evaporated, or decanted dust samples (Supplementary Table S1). To our knowledge, this is the first time that such many dust samples from a single dust event have been collected and analysed with multiple analytical tools. Previously, a total of 136 citizen science and researcher samples from the Pyrenees and French and Swiss Alps have been gained after a major dust event on 4–8 February 2021^23^.

Samples were received from as far north as Kokkola and Oulainen (> 63.8°N) but sample distribution is far from uniform. The densest coverage defines a NW–SE trending corridor in southwestern Finland although it should be noted that there is a lack of samples from the southwestern coastal region of Turku and Rauma (Fig. 7). The most southerly sample received in our citizen sample campaign was from Saarenmaa, Estonia. Processed samples from the Askola region (60.53°N), about 20 km inland from the south coast, record dust deposition of ~ 1100 mg/m^2^. Dust accumulation may vary greatly depending on the location and the amount of accompanying precipitation. For example, at Hartola (61.62 °N), about 168 km northwest from Helsinki, a dust deposition value of ~ 240 mg/m^2^ was recorded.

### Particle properties

#### Magnetic particle characterization

**Figure 8:**
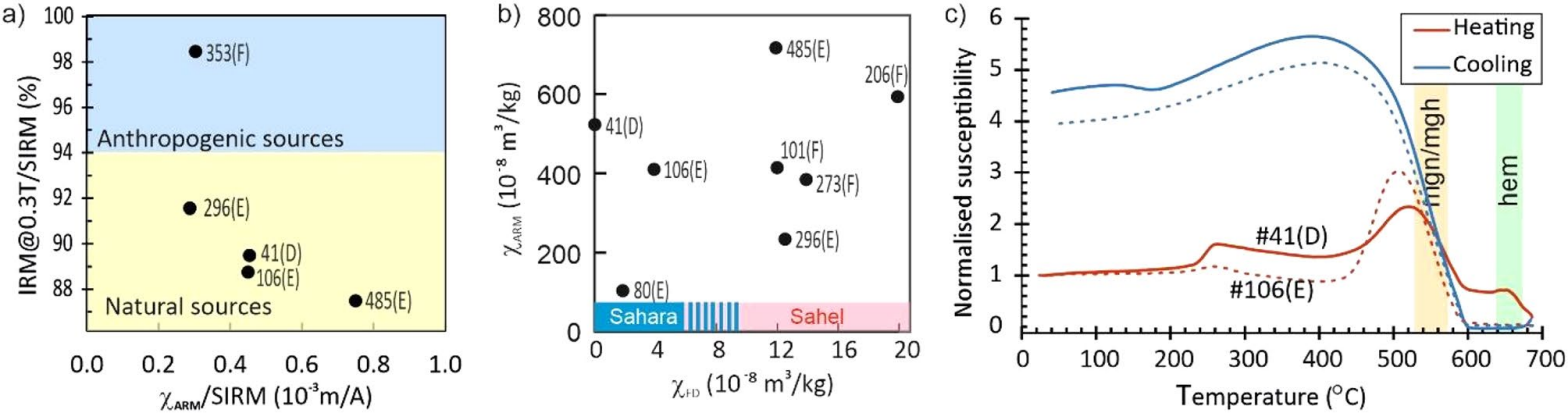
Magnetic particle characterization. (**a**) Ratio of acquisition of isothermal remanent magnetization at 0.3 T and saturation isothermal remanent magnetization (IRM@0.3T/SIRM) versus ratio of ARM susceptibility (χARM) and SIRM (χARM/SIRM). IRM@0.3T/SIRM values for anthropogenic deposition (blue shaded area) and deposition from natural sources (yellow shaded area) from^55^ and χARM/SIRM values for anthropogenic deposition from^56^. (**b**) Values of frequency dependent susceptibility (χFD) vs. ARM susceptibility (χARM), χFD values for Sahara and Sahel dust from^35^. Partly overlapping χFD values for Sahel and Sahara are indicated with blue and pink stripes. (**c**) Thermomagnetic analysis showing temperature dependence of normalised susceptibility (K(T)/K(20 °C). Citizen samples 41 and 106 are indicated with solid and dashed lines respectively. Yellow and green bars indicate the range of Curie and Neél temperatures obtained respectively for magnetite/maghemite, mgn/mgh (hematite, hem) from all of the analysed citizen samples. Letters D, E, F refer to the sampling methods: D = decanted; E = evaporated, F = filtered).

As low-coercivity ferrimagnetic magnetite/maghemite is saturated below 0.3 T and high-coercivity hematite requires larger magnetic field up to ~ 3 T^24^ the acquisition of isothermal remanent magnetization (IRM) analyses show the presence of magnetite/maghemite and minor hematite in bulk dust samples (Fig. 8a). The estimated minimum content of magnetite range from ~ 0.01 to 0.02% and minimum content of hematite range from ~ 0.08 to 0.60%, but we note that the hematite contribution is overwhelmed by co-occuring magnetite (^24^, see “Methods”). In addition, the samples can be divided into two groups based on the frequency dependent susceptibilities (χ_FD_); the first group displays χ_FD_ values < 4 × 10^–8^ m^3^/kg, whereas the second group displays χ_FD_ values > 10 × 10^–8^ m^3^/kg (Fig. 8b). This demonstrates the presence of a significantly higher proportion of nanosized superparamagnetic magnetite or maghemite grains in the second group. Furthermore, samples from the second group also have higher abundances of small single-domain ferrimagnetic minerals, deduced from them on higher χ_ARM_ values (Fig. 8b). Thermomagnetic analyses of the six bulk dust samples show in general similar results (Fig. 8c). The heating curves are characterised by a slight increase in the temperature range of 230–250 °C followed by a prominent peak around 510–520 °C. The Curie points of this phase range between 530 and 560 °C in all samples, indicating the presence of non-stoichiometric magnetite or maghemite. In addition, a decrease of susceptibility in the heating curves was obtained in the range of 650–655 °C, supporting the presence of hematite obtained during IRM analyses as well. Cooling curves show the formation of magnetite/maghemite during the experiment and are therefore irreversible.

#### Mineral composition of the dust samples

**Figure 9:**
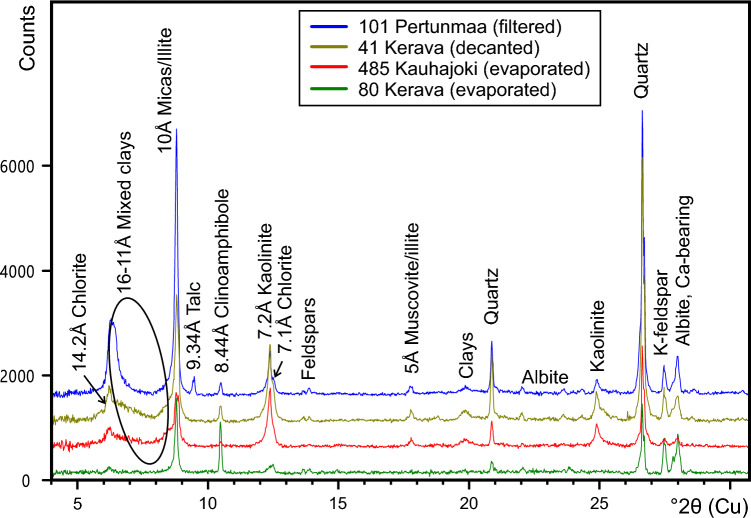
XRD powder patterns of four dust samples reveal mineral compositions dominated by quartz and 10Å micas/illite with minor other clay minerals, feldspars and clinoamphibole. In the vertical count scale, sample 80 is correctly placed and the other samples are displaced by + 500 count steps for comparison.

X-ray powder diffraction data of four dust samples reveal their similar mineral phase composition, dominated by quartz, feldspars, and soft phyllosilicates such as micas and clay minerals (Fig. 9); relative proportions of these phases vary between samples. Sample 101 from Pertunmaa is dominated by quartz and 10 Å mica (biotite/muscovite), although it also has a large 14 Å clay signal (smectite/vermiculite/mixed layer clays). Minor albitic plagioclase feldspar, K-feldspar (likely intermediate microcline), kaolinite, chlorite, talc and clinoamphibole (tremolite-actinolite/hornblende) are also present. Sample 41 from Kerava is relatively enriched in kaolinite clay compared to sample 101. Sample 485 from Kauhajoki contains much less quartz and feldspars than the other samples, and the clay fraction is dominated by illite and kaolinite. The second sample from Kerava, number 80, is distinct from the others in having a very limited clay mineral signal and equal amounts of quartz, albitic plagioclase and K-feldspar. The main clinoamphibole peak at 8.44 Å is also higher in sample 80 than in the other samples. For clinoamphiboles, only the prismatic (110) cleavage reflection at 8.44 Å is visible, implying the presence of asbestiform particles. The yellowish ochre colour of the dust samples implies the presence of goethite/hematite pigment, but this could not be confirmed in the XRD patterns, suggesting either very low abundances and/or poor crystallinity (< 1 wt% detection limit). The distinct lack of signals at the main peak positions for calcite, dolomite and gypsum is also noteworthy.

#### Colorimetry

L* values vary between about 48 and 65 and yield a lightness close to the pale side of the L*a*b* system colour sphere; a* is between 2.4 and 7.1 and b* between 8.1 and 19 (Supplementary Table S2). The highest a* values (redness) are observed for samples 41 (Kerava), 106 (Vantaa), 206 (Hartola), and 219 (Lapinjärvi). The first derivative curves of the reflectance display significant peaks at around 440 nm and 560–570 nm in the measured samples (Supplementary Fig. S1). The first peak indicates the presence of goethite and/or clay minerals, whilst the second peak represents hematite^25,26^.

#### Volume-based particle grain size distributions (GSD)

**Figure 10:**
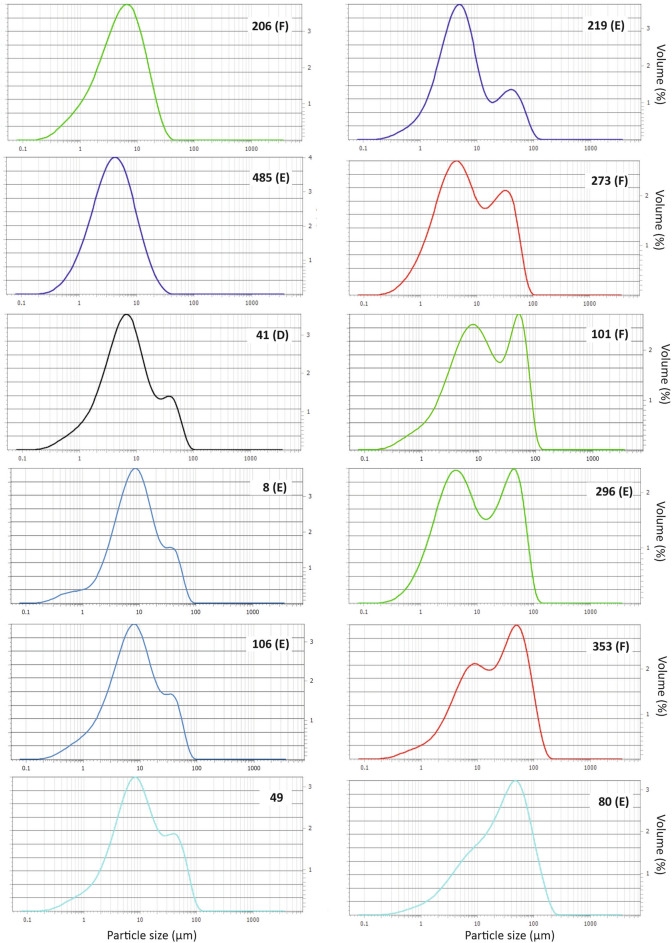
The particle grain size distributions (GSD) of the analysed citizen samples. Data presented as volume (%) per particle size [µm]. Letters D, E, F refer to the sampling methods: D = decanted; E = evaporated, F = filtered).

The median diameters of the dust particles (Fig. 10) fall mostly into the range between 3.9 μm (sample 485) and 9.4 μm (sample 49), with primary modal values ranging typically from 4 µm (samples 273 and 485) to 8.5 µm (sample 8). Most samples demonstrate a bimodal grain size spectrum with a notable secondary mode on the coarse side (ca 30–40 µm). Sample 80 lacks the fine mode and is dominated by significantly coarser particles (median 27 µm and modal value ca 50 µm). This sample is also the least sorted and has the widest range in particle size, with coarsest particles being nearly 250 µm. Samples 206 and 485, collected immediately after the dust event, exhibit a relatively well-sorted unimodal distribution.

#### Volume size distributions of particles < 10 µm

**Figure 11:**
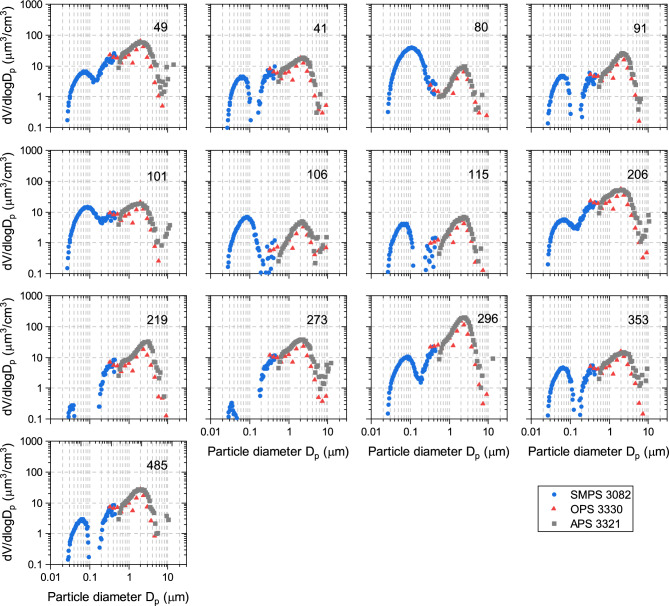
Volume distributions for small particle sizes < 10 µm. Distributions are a composite of measurements using SMPS, OPS and APS.

The volume (or mass) distributions show three modes with varying contributions for different samples, e.g., there is an order of magnitude less volume in the > 1 µm size fraction in sample 80 than in sample 49 (Fig. 11).

#### Comparison of modelled and measured particle sizes

**Figure 12:**
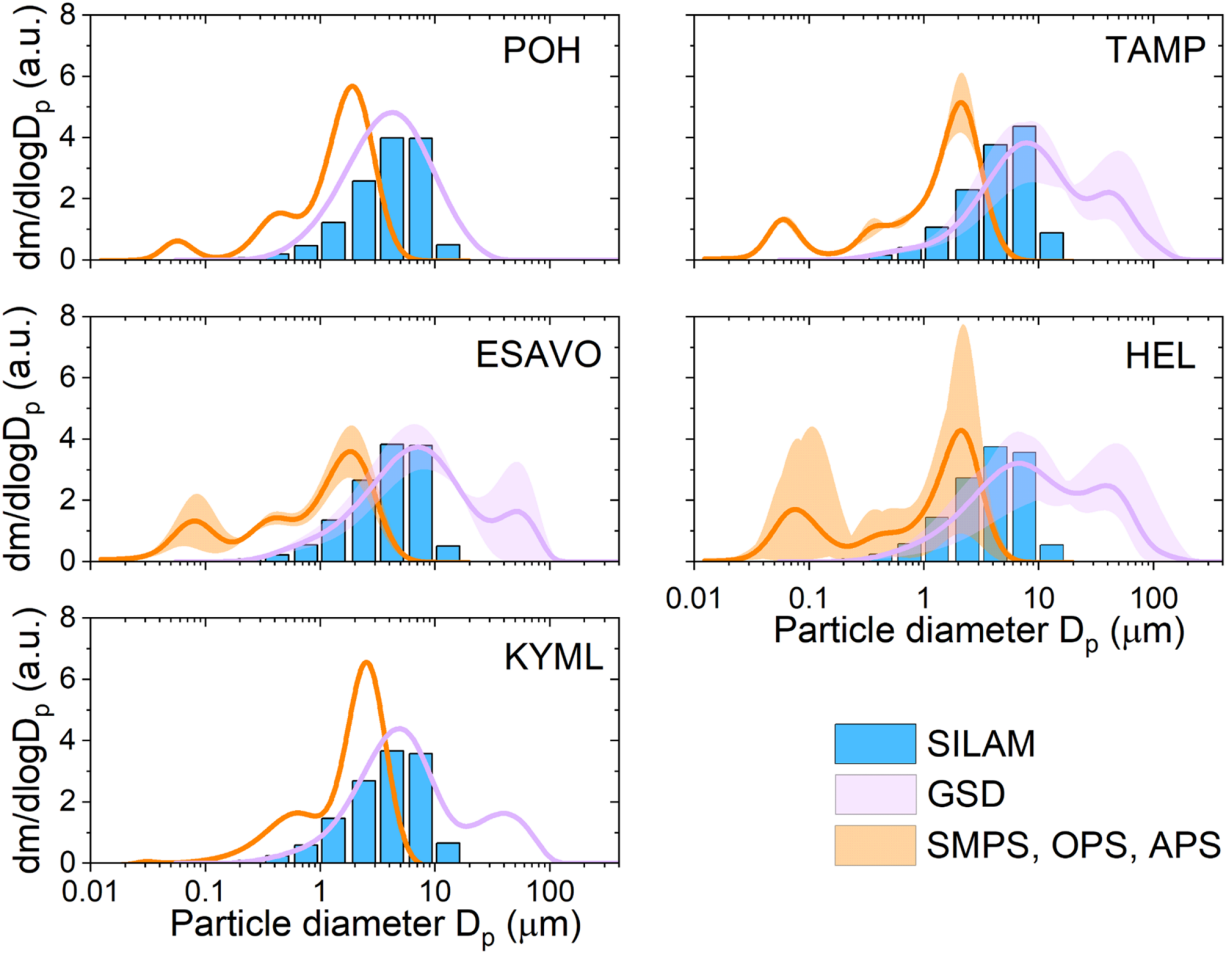
Comparison of wet deposition mass size distribution modelled using SILAM (blue) and measured by GSD (lavender) and SMPS/OPS/APS (orange). Datasets are for the five specified regions: POH (Pohjanmaa, west coast, sample 485), TAMP (Tampere, samples 8, 14a, 91, 358), ESAVO (southern Savo region, samples 101, 206), HEL (Helsinki region, samples 41, 49, 80, 106, 115, 272, 296) and KYML (Kymenlaakso, sample 219). Data presented as dm/dlogDp.

**Figure 13:**
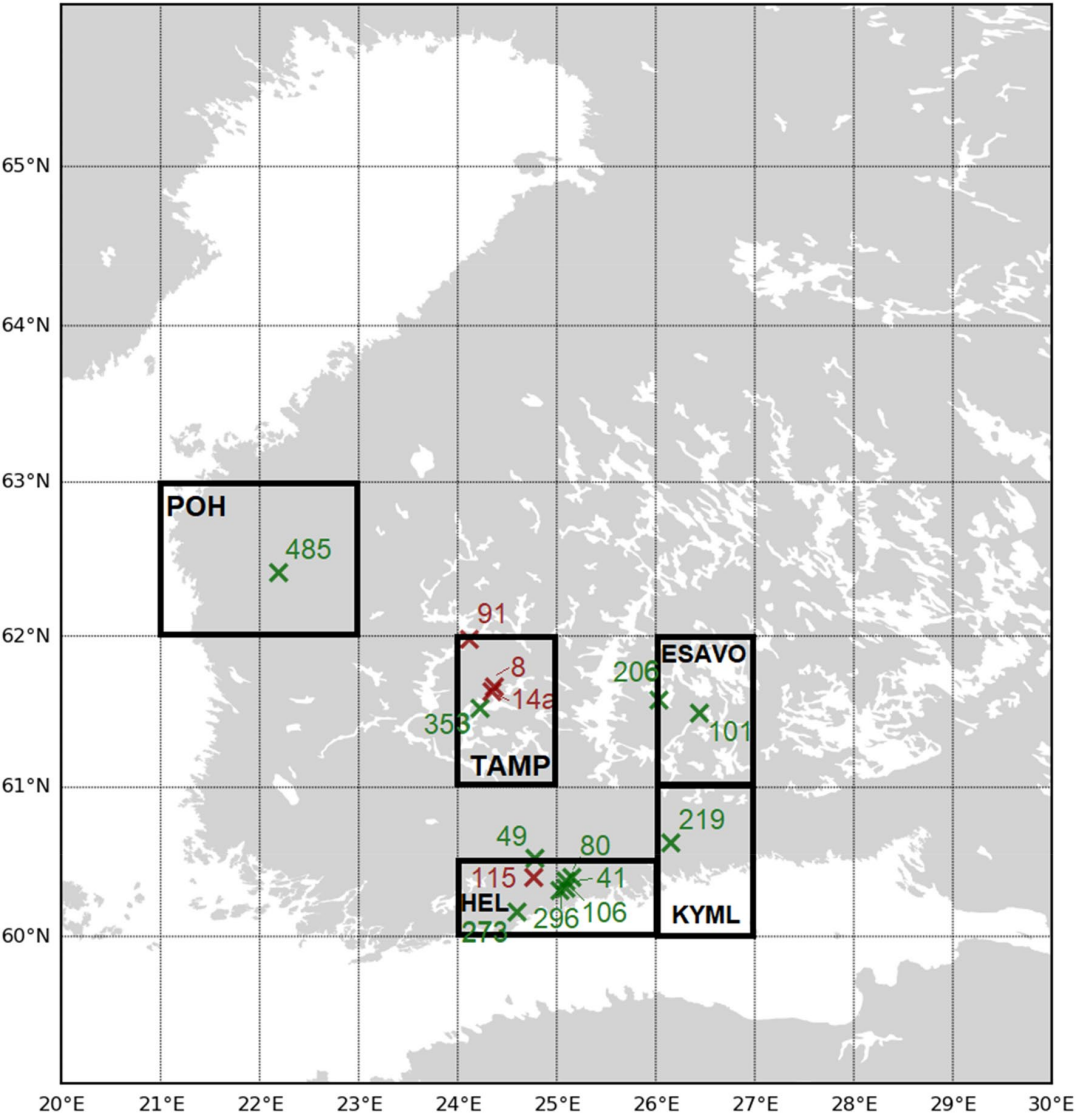
Geographical regions labelled POH (Pohjanmaa), TAMP (Tampere), ESAVO (South-Savo), HEL (Helsinki), and KYML (Kymenlaakso), as used for SILAM and measured particle size comparisons. The green crosses (X) indicate samples and their numbers analysed with two methods, while red crosses (X) with sample numbers show locations where only one of the methods was used. The map was made in Python 3.9.15, using basemap 1.2.1, https://matplotlib.org/basemap/index.html.

When comparing the modelled and measured mass size distributions presented in Figs. 5, 10, and 11, for five specified regions (Fig. 13 shown in Methods), the SILAM model agreed well with the GSD data, which show the pre-treated mineral dust mass distributions (Fig. 12). The SMPS, OPS, and APS measured size distributions used untreated samples. They show a larger accumulation mode (0.1 µm < Dp < 1 µm) fraction than the SILAM or GSD data.

#### Ancillary data

Satellite data indicated that African dust had reached northern Germany and the southern part of the Baltic Sea by 22 February. At this time, all of Fennoscandia was covered by clouds or snow, which prevented the detection of aerosols from space. CALIPSO data above Finland were available for two time slots, but the dust layer view from CALIPSO was blocked by cloud layer data (Supplementary Fig. S3). CALIPSO data show the presence of aerosols below 5 km in the source region on 21 February (Supplementary Fig. S4). Ancillary data on water soluble ions analysed from citizen samples are presented in Supplementary Table S3. FMI hourly automatic weather station (AWS) snow depth data on 21–23 February indicated 19–26 cm snow in the southwestern Finland for Turku Artukainen (60.45 °N, 22.19 °E) and 15–25 cm for the west coast in Rauma Pyynpää (61.14 °N, 21.51 °E), and along the southern coast, 28–33 cm in Espoo Tapiola (60.18 °N, 24.81 °E).

## Discussion

### Development of the episode and model-measurement comparison

Analysis of the 21–23 February 2021 Saharan dust event in Finland shows that the geographical distribution of the wet deposition predicted by SILAM (Fig. 5), and the precipitation patterns simulated with FMI’s operational weather model (Fig. 3), are in good agreement with the observations of dust on snow reported by citizens (Fig. 7). Further analysis reveals that the geographical distribution of dust deposition was far from uniform, with the pattern of deposition coinciding well with the presence of precipitation, therefore favouring scavenging as the deposition mechanism. That hypothesis is also in agreement with the SILAM results. FMI’s HARMONIE-AROME precipitation forecast for 23 February 2021 also agrees with the absence of dust deposition in the observations and the observed minimal precipitation at this time in southwestern Finland. The amounts of deposition calculated from the citizen samples were found to be up to 1.1 g/m^2^ and such maximum amounts per unit area agree with the SILAM calculations.

SILAM source inversion suggests that an extensive area in Africa was the source region for the dust deposited in Finland. The synoptic analysis combined with SILAM results indicates that dust deposited on 23 February 2021 in southern Finland was transported directly from North Africa via a strong south-westerly flow at an altitude just below 5 km. Freezing rain and snow in the frontal system accompanying a low-pressure system that developed over the Baltic Sea, resulted in scavenging of dust, thus explaining the brownish-yellow colour of snow in southern Finland. The SILAM source inversion (Fig. 6) as well as the measurements of the magnetic properties of the particles (Fig. 8b) are in accordance with MODIS satellite data of NASA from 21 February 2021 (https://worldview.earthdata.nasa.gov/?v=-42.68097473875406,-4.833863288662396,44.67745998325363,44.0777603187117&t=2021-02-21-T09%3A07%3A36Z), which suggest that the dust originated from a large area in the western half of Sahara^27^. As far as we know, there are no reports of deposition from this dust event in proximity to the source area. It may also be noted that polar jets and associated atmospheric circulation have previously been identified as capable of bringing dust from Sahara to the Arctic^28^.

### Modelled and measured particle sizes

The comparison of modelled and measured mass size distributions shows that SILAM matches well with GSD measurements in the size mode < 10 µm, while underpredicting the abundance of particle mass in the mode > 10 µm. The peak in a giant particle size range (> 20 µm) registered in Southern and Central Finland is not reproduced. An additional analysis, e.g., scanning electron microscopy, combined with single-grain geochemistry, would be needed to confirm the origin of these coarse particles. Their transport from the Sahara desert seems improbable due to fast removal of so coarse particles via sedimentation. This phenomenon, however, is currently under discussion because models would require major reparameterization of deposition processes to reproduce such observations^57^. The effects considered this-far (e.g., particle asphericity) do not explain the magnitude of the effect^29–31^. Investigating SDS events through citizen science observations has great potential for providing additional evidence.

The GSD measurements are best suited for particles with sizes larger than 0.1 µm. A significant mass contribution of smaller size mode particles was observed for untreated samples in the SMPS, OPS, APS data. These peaks correspond well to the urban-aerosol size range (~ 0.1 µm). Compared to GSD, the OPS and APS are not sensitive to particles > 3 µm, either due to line losses or larger particles not being atomized with the TSI atomizer. However, the mass distributions measured with the combination of SMPS, OPS, APS (Fig. 10) are comparable to dust size distributions measured in Cape Verde^32^. The untreated citizen-collected samples from close locations showed considerable differences in the SMPS, OPS, APS data, but quite consistent distributions after the pre-treatment (GSD data). This suggested that at a local scale, there was a varying degree of contamination by soluble components, whereas the mineral dust fraction of particles was similar.

### Outcome of the citizen-science project

The magnetic analyses of citizen samples show the presence of low coercivity ferrimagnetic magnetite/maghemite (minimum content ~ 0.01–0.02%) and high coercivity antiferromagnetic hematite (minimum content ~ 0.08–0.60%), in the bulk dust samples, which is typical for Saharan dust^33^. This was corroborated by the colour reflectance analyses indicating that hematite was the dominant contributor to the reddish colour of the samples. The Saharan dust deposited on the snow in Poland in 2021 yields a similar thermomagnetic signal but lacks the diagnostic indications of hematite^34^. Goethite was not detected in the magnetic studies nor in the XRD analyses. Therefore, the 440 nm peak detected in the colorimetry likely indicates the presence of clay minerals only. The group of samples with χ_FD_ values > 10 × 10^–8^ m^3^/kg is interpreted to have higher concentrations of fine-grained superparamagnetic ferrimagnetic minerals than the other group with lower values. Dust sources have been discriminated between the arid Saharan and the more humid Sahel sites based on the concentration of fine-grained ferrimagnetic minerals, suggesting that magnetic properties reflect differences in weathering regime of the source material^35^. Higher rainfall at the Sahel promotes more intense weathering and pedogenesis thus producing more fine-grained ferrimagnetic minerals and therefore bulk dust samples from the Sahel showed high χ_FD_ values (> 5 × 10^–8^ m^3^/kg)^35^; (Fig. 9b).

The XRD analysis of five bulk dust samples identified mineral phases that correspond well with atmospheric mineral dust in general^36^ and with dust typical of north Africa (e.g.^37^), containing quartz, feldspars (plagioclase, K-feldspar), clinoamphibole (tremolite, hornblende), micas (biotite, muscovite), clay minerals (illite, kaolinite, chlorite and smectite) and traces of talc in one sample. The relatively high content of clay minerals correlates well with their known relative enrichment by long distance transport^38^. These all belong to the silicate group of minerals which are also typical of soils derived from Precambrian bedrock, which is widespread both in Finland and the western Sahara. One important observation is that carbonate minerals (calcite, dolomite) and gypsum are missing. For example, Moroccan dust is reported to be particularly rich in carbonates^36^. It is possible that carbonate particles have been destroyed by atmospheric processes^39^, consistent with our finding of high Ca^2+^ concentration in the IC results (Supplementary Table S3). These would have provided a clear indication of a remote provenance, as they are not locally available. Quartz and alkaline (Na–K) feldspars are typical of granitic or metamorphic primary sources or their sedimentary derivatives, but chlorite, clinoamphibole and talc indicate derivation from low to intermediate metamorphic environment and more mafic (Mg-Fe-rich) source rocks than granitoids.

Scavenging and mixing of local dust with material derived from distant sources could explain some of the different mineralogical characteristics between samples. It is important to note that citizen samples were prepared by three different methods, filtering, evaporating, or decanting, and the method of choice may affect the particle sizes and mineral composition of the samples. Nevertheless, we found that all methods, even filtering, still retain the smallest particle sizes, including clay and magnetic nano-particles. Moreover, our findings suggest that delays in collecting samples may have affected some of the particle properties. This might explain, e.g., why one of the samples from Kerava (80, collected 1 March, as one of the latest collected samples) contained a smaller clay and fine silt fraction than the others. In addition to the granulometric properties, the Kerava sample yielded distinguishable characteristics compared to the remaining samples, specifically the absence of clay minerals in the XRD analysis, least amount of reddish hue, and distinct magnetic properties. Rain might cause the same effect on the snow surface, by washing away the fine clay fraction and soluble salts; considering the duration between deposition and sample acquisition, contamination from nearby sedimentary sources is also likely.

When dust is deposited on snowpacks and identified only later, the results for water soluble ions can be insightful. In^40^ the threshold value for identifying the presence of Saharan dust in alpine snowpacks close to the Sonnblick Observatory was defined as having a pH > 5.6 together with a Ca^2+^ concentration > 10 µg/l. Interaction with sea salt during the transportation, anthropogenic sources, precipitation and melting of snow and sample contamination may have influenced some of the observed water-soluble ion contents in the analysed citizen samples.

The citizen-science part of the study complemented other observations and provided large amount of data when other tools were inefficient. On 23 February, satellite imaging over Central Finland was obscured by clouds, whereas deposition intensity varied greatly from place to place following the precipitation intensity and dust amount in the air. For such conditions, citizen observations were the only way to verify where and how much dust was deposited. Based on our experience, we argue that citizen science can greatly contribute to improve our understanding of dust events and to complement regular in situ and satellite observations on dust.

As a general requirement, when citizens are asked to collect dust on snow samples, it would be useful to have a set of instructions to ensure that sample quality is as uniform as possible and that samples are collected as soon as possible, and certainly within one week of the depositional event. We suggest the following sampling procedure for any future dust event deposited on snow: to best preserve all the particle sizes, snow should be evaporated, and the remaining particles should not be moved from the container in which the evaporation took place. To preserve the magnetic properties of the samples, the particles should not be subjected to temperatures higher than 90 °C. Therefore, we suggest placing the dusty snow sample on a sheet of aluminium folio on an oven tray and evaporating the snow in the oven at a temperature of < 75 °C temperature. When dry, the foil sheet should be carefully folded and labelled, before sending to the responsible research institute.

## Conclusions

A rare event of massive African dust transport towards Finland and eventual scavenging of the dust from altitude of about 4 km occurred on 21–23 February, 2021. The present investigation aimed at utilizing citizen science to verify African dust transport and deposition modelling results of the event. It was shown that the episode was successfully forecasted by meteorological and dispersion models and reproduced with a more detailed retrospective analysis.

The largest known set of citizen samples of dust from 525 locations over a wide territory in southern Finland was gained. Citizen samples revealed a quite detailed picture of the event and also served for evaluation of the model predictions.

The map of wet deposition during the event predicted by the SILAM model was found to be in good agreement with the observations of dust on snow reported by citizens. The pattern of deposition coincided well with the presence of precipitation, suggesting scavenging as the deposition mechanism, also in agreement with the SILAM results. The amounts of deposition were up to 1.1 g/m^2^. An extensive area in Africa was shown to be the source region for the dust, based on modelling and on the characteristics of fine-grained ferrimagnetic minerals of the samples.

The comparison of modelled and measured mass size distributions revealed that SILAM matched well with GSD measurements in the size range up to 10 μm, while underpredicting the abundance of particle mass in the giant size range. The colour reflectance analyses indicated that hematite was the dominant contributor to the reddish colour of the samples, and the identified mineral phases correspond well with dust typical of north Africa.

In summary, our study demonstrated that citizen science can potentially be used to fill in gaps in data collection and so help to assess the status and impacts of dust events in Finland, and elsewhere. Citizen engagement also raises public awareness and increases action in monitoring changes in our environment and promotes interest in environmental research. Finland is an excellent place for studying post-depositional dust-snow interactions, as seasonal snow, combined with flat terrain and the occurrence of five global snow classes out of six, creates ideal conditions for natural experiments.

## Methods

### SILAM modelling

The System for Integrated modeLling of Atmospheric coMposition, SILAM, is a global-to-local scale atmospheric composition model (http://silam.fmi.fi^19^). The model has been designed for a wide range of tasks including traditional AQ predictions, emergency applications, data assimilation, and source apportionment studies. Relevant for the current case, turbulent vertical exchange follows the Monin–Obukhov formalis^41^, implemented via extended resistance analogy scheme^42,43^. SILAM is based on Carbon Bond 05 (CB05) chemistry^44^ extended with stratospheric reactions, secondary aerosol formation and sulphur chemistry. A more detailed description of the model chemistry has been presented in^44,58^, except that currently CB05 is used instead of Carbon Bond 4. Dust is not impacted by model chemistry. The source apportionment techniques include 4-D variational assimilation 4D-VAR^44^ and Ensemble Kalman filter^45,59^. A simplified procedure of source delineation via footprint analysis (in-essence, the first iteration of 4D-VAR) has been applied to analysis of snow samples^46^.

The current event was simulated by SILAM twice. The first simulation was the operational automatic dust forecast (http://silam.fmi.fi) performed with a resolution of 0.1° × 0.1° over the globe, driven by the meteorological fields of the Integrated Forecasting System (IFS) model of the European Centre for Medium-Range Weather Forecast (ECMWF). The key output variable is a concentration within the bottom-most model layer (~ 0–25 m). The forecast temporal span is 5 days forward. The operational forecasts calculate the deposition rates as an output variable, but these are not archived. The second limitation of the operational setup is a limited number of dust size bins–4, up to 20 µm.

To perform a more detailed analysis, dedicated simulations were performed, with 13 bins (0.1–0.18 μm, 0.18–0.32 μm, 0.32–0.56 μm, 0.56–1.0 μm, 1.0–1.8 μm, 1.8–3.2 μm, 3.2–5.6 μm, 5.6–10 μm, 10–18 μm, 18–32 μm, 32–56 μm, 56–100 μm, and 100–180 μm) (representing the dust size distribution and the optical column depth and deposition fluxes stored in the output files. The spatial resolution was kept at 0.1°.

### Aerosols in HARMONIE-AROME weather model

Near-real-time aerosol data from Copernicus Atmosphere Monitoring Service (https://atmosphere.copernicus.eu) are being introduced to a development version of HARMONIE-AROME. In this study, very first test results were shortly mentioned. In the reference version of HARMONIE-AROME^20,21^, climatological data on aerosol optical depth at 550 nm, based on^60^ are applied only for radiation parametrizations. Prescribed constant values of the number of cloud condensation nuclei (separate over land and sea) are assumed when deriving liquid droplet number concentration and assuming effective radius of cloud droplets for the default radiation scheme. The mass mixing ratio (MMR) of aerosol species considered in the development version of HARMONIE-AROME includes sea salt (3 size bins), sulfate, desert dust (3 size bins), black carbon, organic matter, nitrate and ammonium. Three-dimensional fields from CAMS forecast are imported via horizontal boundaries to the initial state of each HARMONIE forecast cycle. During the forecast, the aerosol fields evolve due to advection, boundary relaxation, possibly due to wet and dry deposition/sources of desert dust and sea salt species. MMRs of hydrophilic and hydrophobic aerosol species are assumed to influence the liquid droplet and ice crystal number concentrations, that are used for parametrization of the specific content (mass) of cloud liquid and ice as well as precipitable rain, snow and graupel. These all are three-dimensional prognostic model variables that convert to precipitation fluxes at the surface via the existing cloud microphysics parametrizations described in^20, 21^. For radiation, MMRs are combined to aerosol inherent optical properties (mass extinction, single-scattering albedo and asymmetry factor that depend on aerosol species, wavelength, humidity), taking into account the humidity in the model, to obtain run-time three-dimensional aerosol optical properties on every gridpoint of the model. Liquid droplet number concentration may be used to derive cloud droplet effective radius for the default radiation parametrizations.

### Citizen science initiative

We use the term citizen science here to refer to the participation of the public in scientific research, through cooperation with scientists utilising social media, mobile devices, the internet, and other, more traditional media. In the dust event reported here, which originated from Africa, dust was deposited in the southern part of Finland on 23 February 2021, extending further inland, at a time when the ground was widely covered with snow, thus making the accumulation of dust more easily detectable. This caused members of the public to contact the FMI to find out what was happening, and what was causing colouring of the snow on the ground, and why orange snow was falling from the sky. These concerns were redirected and responded to quickly by two scientists at FMI, with the help of social media^47,48^. These responses initiated a citizen science project on Saharan dust, which contained information on the Saharan SDS event reaching Finland, appeared on the internet in the front page of the FMI website and the FMI TV meteorologists announced, as part of the national television weather forecast, the invitation to collect and send dust samples to FMI for research. The information and instructions soon spread via social media and the internet, as well as in other media, including newspapers, magazines, television, and radio. People were asked to report their observations on Saharan dust, including information on the location, time, and dust colour in various tones of yellow, brown, and red. Most importantly, people were asked to collect dust-containing snow, and through filtering, evaporating, or decanting, to extract the dust according to the guidelines provided^47,48^, and then to send them to FMI for further investigation. Overall, the event received considerable national attention and resulted in samples being collected by citizens from 525 locations, with one to more than ten samples being included in each submitted letter.

### Citizen samples

Most citizen samples consisted of a solid residue from 2 dl of superficial snow, that had been either melted and filtered using coffee filters, evaporated on aluminium foil, or decanted with the help of containers. Coffee filters were used to extract the solid dust residue from the snow samples, as coffee filters are commonly found in every household in Finland. Citizens were asked to place the snow sample in a coffee filter on top of a container letting the melted snow filter through the cellulose fibers, retaining the solid residue in the inner surface of the filter. Melting of the snow sample could be done prior to the filtering. The sample-containing coffee filters should be left to fully dry before sending them to FMI for their analysis. Each of the citizen samples and accompanying information were processed by giving a sample identification number, quality flag and map location, before being archived within a digital database. The samples and related correspondence were organised in storage boxes located at FMI. All of the original information provided by the citizens was translated from Finnish to English into a database. The citizen samples represented latitudes 60–64.3°N, while one sample came from Estonia. One additional citizen sample that had been collected earlier from Timbuktu, Mali, southern Sahara, was used as a reference sample for IC analysis and light microscopy. In addition to citizen samples, researchers also collected dusty and non-dusty snow samples which were stored in a freezer for further analysis. Individual samples that were used for investigating the particle properties are listed in Supplementary Table S1. The samples from 525 locations were quality flagged on the basis of the sample amount and on the method used for separating the particles from the snow, including filtering, evaporation and decanting. Representative samples were selected to characterize the accumulated dust. For the grain size distributions (Fig. 10), 12 samples were utilized based on their quality and the amount of particles available for the analysis, and on their location on the modeled precipitation type and deposition particle size and amount map (Figs. 5 and 13). For volume distributions for small particles (Fig. 11), 13 samples were utilized. The geographical regions of Fig. 13 were used based on modeled precipitation (rain, freezing rain and drizzle, sleet, graupel and snow were all suggested over different parts of the forecast domain (Fig. 3a), highlighting the difference for the southern coast region HEL) and deposition particle sizes (Fig. 5b highlighting a potentially larger deposition for ESAVO; and Fig. 5b for KYML, TAMP, POH, where POH represents the northernmost deposition). Samples selected for the magnetic properties (11 samples) and XRD (4 samples) represent different sampling methods and geographical locations. The sampling methods (decanted, filtered, evaporated) are indicated in the corresponding figures (Figs. 8, 9 and 10).

### Colorimetry

Colorimetry was measured using a Konica Minolta CM-26d spectrophotometer, set on a 3 mm target radius and recorded in L*a*b* colour space (lightness L*, from 0 (black) to 100 (white); a*, a green–red continuum, positive values associated with red and negative values with green; and b*, a blue-yellow continuum, positive values associated with yellow and negative with blue). The spectrophotometer was calibrated at the start of each set of measurements with a zero (black) and white standards, and was set to take three sequential measurements, giving the mean values of the obtained colour coordinates. Measurements were conducted with reference illuminant CIE D65, observer angle 10° and 54 mm integrating sphere. The spectral reflectance was measured from 360 to 740 nm with 10 nm resolution.

### Magnetic particle characterization

Bulk dust samples packed to standard 8 cm^3^ diamagnetic cubes were used for all except the thermomagnetic measurements. Analysed samples are indicated in the Supplementary Table S1 (citizen samples). Low frequency (0.5 kHz) magnetic susceptibility (χ_LF_) and high frequency (4 kHz) magnetic susceptibility (χ_HF_), anhysteretic remanent magnetisation (ARM), susceptibility of ARM (χ_*ARM*_), acquisition of isothermal remanent magnetisation (IRM) and saturation isothermal remanent magnetization (SIRM) at 2 T were carried out at the Solid Earth Geophysics laboratory of the University of Helsinki. Thermomagnetic measurements were done at the Geophysical Laboratory of Geological Survey of Finland. Trace quantities of magnetic minerals can be detected with magnetic methods (e.g. magnetite < 1 ppb and hematite < 10 ppm).

Mass-normalized low and high frequency magnetic susceptibility measurements were carried out using SM100 (ZH Instruments) in a field of 320 A/m to assess bulk magnetic mineral concentration. The measurements at the two frequencies were used to calculate the frequency dependent susceptibility (χ_FD_) (χ_FD_ = χ_LF_ – χ_HF_), which also can be defined as [χ_FD_ % = (χ_LF_ – χ_HF_)/χ_LF_ × 100 (%)]. The loss of susceptibility between the two frequencies reflects the response of nanosized superparamagnetic (SP) grains, higher values suggest a larger amount of these grains^49^ SP grains are known to form during weathering and pedogenesis^52^. Anhysteretic remanences were imparted using AGICO LDA-3A AF demagnetizer with AMU-1A magnetizer in a 100 mT peak alternating field (AF) and a steady direct current biassing field of 0.05 mT. ARM was measured using a 2G SQUID magnetometer and samples were demagnetized in a stepwise manner with 12 increasing steps from 2.5 mT to 100 mT. The ARM susceptibility* (*χ_ARM_*)* was calculated by dividing the ARM by the biassing field. ARM an*d* χ_ARM_ are sensitive to the concentration of small ferrimagnetic minerals in the size range of single-domain (SD), because these are efficient at acquiring remanence^50^. In particular, nanosized superparamagnetic (SP) grains are supposed not to carry remanence^50^. *S*tepwise acquisition of isothermal remanent magnetization (IRM) in fields from 20 mT up to 2 T was performed on six of the samples using an MMPM10 pulse magnetizer and for five samples, only a 2 T isothermal remanent magnetization was imparted. Remanence induced with the 2 T field is called a saturation isothermal magnetization (SIRM), even though the field of 2 T is sufficient to saturate ferrimagnetic magnetite and maghemite but not antiferromagnetic hematite or goethite. Magnetization was measured with a 2G SQUID magnetometer. Samples were demagnetized with the same 12 increasing steps as ARM. SIRM is an indicator of the concentration of magnetic minerals in a sample but also responds to magnetic grain size^61^. A low coercivity mineral (ferrimagnetic: magnetite, maghemite) will acquire remanence at low fields and a high coercivity mineral (antiferromagnetic: hematite, goethite) will magnetize only at high fields. χ, ARM and SIRM are dependent on the concentration of magnetic minerals. Ferrimagnetic material (magnetite, maghemite) have a higher intrinsic magnetization than antiferromagnetic material (hematite, goethite), and thus most parameters dependent on concentration typically reflect changes in ferrimagnetic minerals^51^. From these measurements a range of diagnostic ratios indicating grain size of ferrimagnetic minerals were calculated. Since SIRM is rather insensitive to variations in grain size, it is common to estimate the relative abundance of SD magnetite grains by the ratio of χ_ARM_/SIRM. Samples containing a higher SD fraction will yield higher values. The χ_ARM_ is strongly size dependent whereas low field susceptibility χ shows the same value over a very wide range of grain sizes. In addition, remanence ratios for IRM acquisition were calculated to indicate the low-coercivity ferrimagnetic ‘soft’ (magnetite, maghemite) and high-coercivity ‘hard’ antiferromagnetic (hematite) minerals. A ‘soft’ mineral will acquire remanence easily, at low fields and a ‘hard’ mineral (e.g. hematite) will magnetize only at high fields (e.g. IRM@0.3 T/SIRM of ~ 30%)^61^. Minimum magnetite content was calculated based on the remanence acquired with IRM field of 0.3 T following^53^) who state that remanence at 0.3 T is half of the saturation of the dispersion of the single-domain magnetite crystal (90–92 Am^2^/kg). As the magnetite is rarely purely stoichiometric and can contain some impurities, this formula gives the estimation for the minimum content of magnetite. Similarly, minimum hematite content was calculated using the remanence acquired between 0.3 and 2 T and the saturation value of 0.4 Am^2^/kg for hematite. However, even small amounts of magnetite co-occur with hematite, its ~ 230× stronger magnetization can overwhelm the magnetic contribution of hematite (e.g.^53^).

Magnetic mineralogy was further investigated by using thermomagnetic analyses on six powdered bulk dust samples. Temperature dependence of low-field magnetic susceptibility was measured from 20 to ~ 700 °C (in argon) followed by cooling back to room temperature using AGICO MFK1-FA kappabridge. During heating, magnetic minerals become paramagnetic and lose their susceptibility at specific temperatures, and some display crystallographic changes that aid in their identification.

### Mineralogical characteristics

X-ray powder diffraction (XRD) phase identification was done for four dust samples at the Geological Survey of Finland (GTK), using a Bruker D8 Discover (A25) powder diffractometer equipped with Cu-anode X-ray tube, motorized divergence slits, motorized anti-scatter screen, sample spinner, and LYNXEYE XE-T detector. Each sample was powdered by manually grinding it with an agate mortar in acetone suspension; the suspended sample was poured on preparation glass, spread equally, and dried. X-ray powder diffractograms were measured for 0.5 h using 40 kV/40 mA generator settings, 4–110° 2Ө range and 0.06°2Θ/s angular velocity. The phase identifications were done using Bruker EVA 6.0 software and ICDD (International Centre for Diffraction Data, Powder Diffraction File) PDF-4 Minerals 2022 database. The diffraction data were treated using background fitting and simulation to fixed slits. Due to the very low and variable sample amount of ~ 50 mg on glass preparation, the primary diffractograms contained a high amorphous background which was extracted.

### Bulk grain-size distribution (GSD)

For grain size analysis, approximately 0.1–0.3 g of bulk sediments was pre-treated with 30% H_2_O_2_ and 10% HCl to remove carbonate and organic material following a standard procedure for grain size analysis (cf.^62^), followed by dispersion using ultrasonication with Na_4_P_2_O_7_⋅10H_2_O. The grain-size distributions for the bulk sediment samples were analysed with a Fritsch A22 Analysette 22 NeXT Nano measuring unit providing grain-size measurements in the range of 0.01–3800 μm.

### Nano size particle distributions (SMPS, OPS, APS)

Suspensions of dust samples in Milli-Q water were dispersed using a constant output atomizer (TSI 3076) and dried with a 1 m long, vertically arranged Nafion dryer (PermaPure DD-600). The dry aerosol flow was subsequently isokinetically split into three separate flows that were measured using a scanning mobility particle sizer (SMPS, TSI 3938), an optical particle sizer (OPS, TSI 3330) and an aerodynamic particle sizer (APS, TSI 3321). The SMPS was used to measure the size range of 0.01–0.4 μm, the OPS that of 0.3–9 μm, and the APS that of 0.5–20 μm. The particle dispersion method and line losses limited the size of aerosolized dust particles to below 8 μm, with significant losses occurring for particles larger than approximately 3 μm.

### Comparison of modelled and measured particle sizes

To compare the measured and modelled size distributions, the data were converted to mass distributions and normalized by the width of the used size bins on a logarithmic scale dm/dlogD (a.u.). The different geographical regions (POH, TAMP, ESAVO, HEL, and KYML) in which datasets are compared are presented and explained in Fig. 13.

### Ancillary methods

FMI AWS snow depth data were utilised with an accuracy of ± 2 cm. Deposited dust per unit area was calculated using citizen samples with adequate metadata. Light microscopy photography was carried out for first estimations of particle properties.

Citizen samples from Finland and a reference soil sample from Timbuktu were used for IC-analysis. Dust was weighed (Mettler Toledo XPE204 Analytical Balance) in test tubes. MilliQ-water was added to each test tube (5 mL for sample 106, 10 mL for all others). The tubes were put on an automated mixer for an hour to dissolve all water-soluble ions. Sample 106 was diluted 1:10 with MQ-water. All other samples were undiluted. The tubes were stirred on an automated mixer for half an hour immediately prior to IC-analysis. From the analysis results, the non-sea salt concentrations of nss-K, nss-Mg and nss-Ca were calculated to estimate how much K, Mg and Ca is contributed from elsewhere. Since K is also derived from biomass burning, Mg and Ca can be considered as tracers for soil particles.

Ensemble forecast for dust optical depth and surface concentration on 21 February 2021, provided by the WMO SDS-WAS NA-ME-E Regional Center, http://sds-was.aemet.es) were used to study the dust emission and transport on 21–23 Feb 2023 (Supplement Video).

Satellite data from NASA MODIS (modis.gsfc.nasa.gov), Worldview (worldview.earthdata.nasa.gov), and NOAA JSTAR Mapper (http://star.nesdis.noaa.gov/jpss/mapper) indicated that African dust reached northern Germany and the south of the Baltic Sea by 22 February. All Fennoscandia was covered by clouds or snow at this time, which prevented detecting aerosols from space above Finland. However, National Aeronautics and Space Administration NASA CALIPSO data over the source area in Africa showed that dust transport occurred at altitudes below the polar jet levels, which show dust clouds below 5 km on 21 Feb. 2021 (Supplementary Fig. S4).

### Supplementary Information

Supplementary Information.

Supplementary Video 1.

## Data Availability

All data needed to evaluate the conclusions in the paper are present in the paper.
