# Supplementary material for: African dust transport and deposition modelling verified through a citizen science campaign in Finland
Source: Sci Rep. 2023 Dec 4;13:21379. doi: 10.1038/s41598-023-46321-7 (PMC10695925; doi:10.1038/s41598-023-46321-7)
Supplement: Supplementary file 1 — Supplementary Information. [file 41598_2023_46321_MOESM1_ESM.docx]

# African dust transport and deposition modelling verified through a citizen science campaign in Finland

Outi Meinander^1^*, Rostislav Kouznetsov^1^, Andreas Uppstu^1^, Mikhail Sofiev^1^, Anu Kaakinen^2^, Johanna Salminen^2,3^, Laura Rontu^4^, André Welti^5^, Diana Francis^6^, Ana A. Piedehierro^5^, Pasi Heikkilä^3^, Enna Heikkinen^1^, Ari Laaksonen^5,7^

**Supplementary Information**

**Supplementary Table S1.** Citizen samples used for investigating particle properties. The locations of all the 525 citizen samples from southern Finland, 60- 64.3 °N were used to verify the spatial variability of the deposition modelling results. Selected samples, based mostly on the quality of the sample and the amount of particles available for the analysis, were used for analysis of particle sizes, deposition amount, color (C), minerals (XRD), magnetic properties (M) and ion chromatography (IC), as indicated in the Table. One additional citizen sample, not from this event, collected from the ground in Timbuktu was used as a reference sample for IC (and investigated as a reference sample under light microscope). Based on magnetic properties, samples identified to originate from Sahara are marked in the table with one asterisk (*) and Sahel with two (**). Crosses (X) indicate the type of analysis carried out with each sample.

| **No. 1-**  **524** | **Location** | **Collection date** | **Collection technique** | **Sample photo** | **Size GSD** | **Size <10 µm** | **Weighted [g]** | **C** | **XRD** | **M** | **IC** |
| --- | --- | --- | --- | --- | --- | --- | --- | --- | --- | --- | --- |
| **8** | 61.67, 24.36  (Orivesi) | 1.3.2021 | Evaporated | 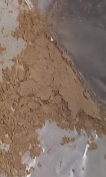 | X |  | X  0.10 |  |  |  |  |
| **12B** | 60.49,  25.71  (Askola) | 3.3.2021 | Evaporated (1m^2^ dusty snow on a frozen pond) | 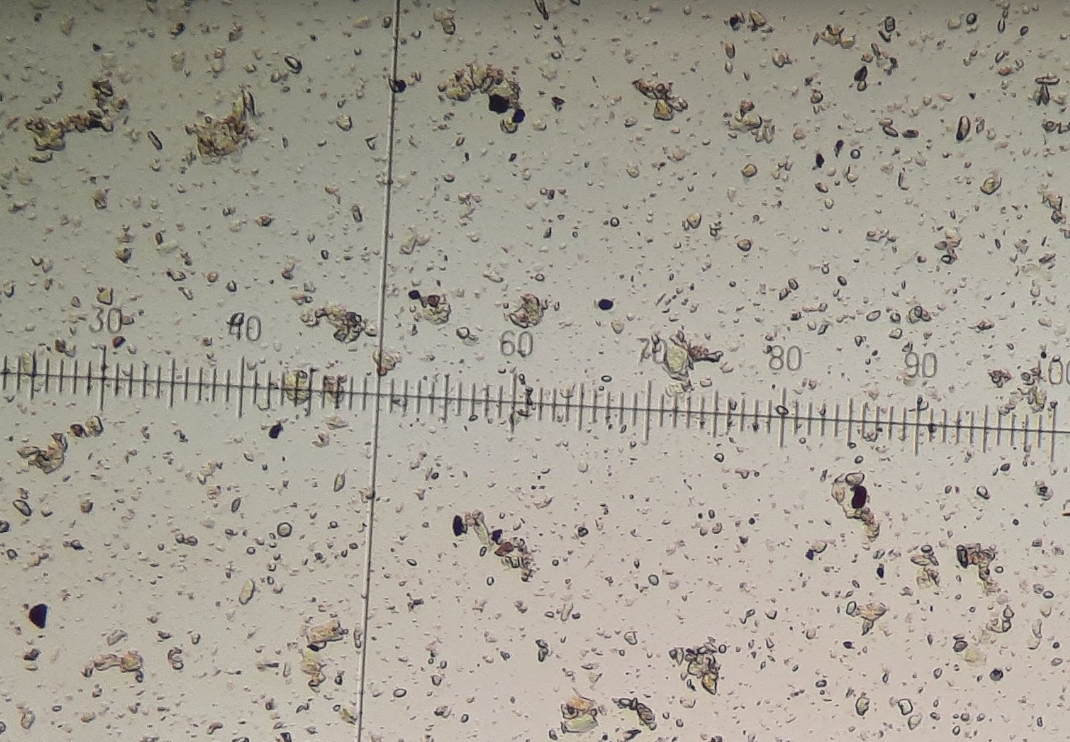 |  |  | X  1.12 |  |  |  |  |
| **14A** | 61.64, 24.35  (Orivesi) | 28.2.2021 | Evaporated (Bottom layer of dust in a bucket) | 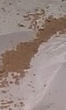 |  | X | X 0.31 |  |  |  |  |
| **41** | 60.37, 25.08  (Kerava) | unknown | Decanted  (30 l snow) | 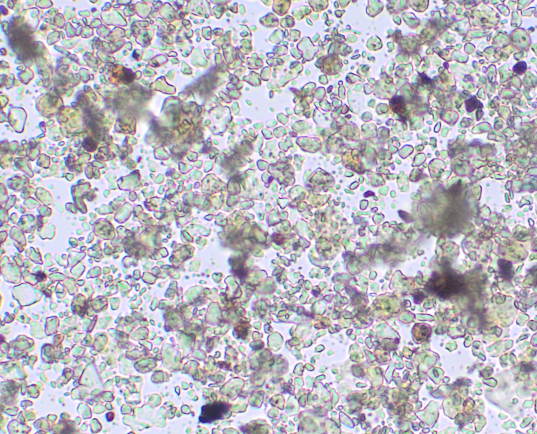 | X | X | X  0.56 | X | X | X  * | X |
| **49** | 60.52, 24.77  (Nurmi-  järvi) | 1.3.2021 | unknown | 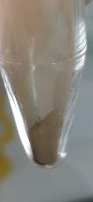 | X | X | X  0.09 | X |  |  |  |
| **80** | 60.40, 25.15  (Kerava) | 1.3.2021 | Evaporated | 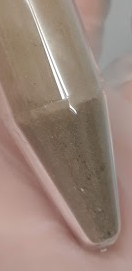 | X | X | X  0.47 | X | X | X  * |  |
| **91** | 61.98,  24.11  (Ruovesi) | 27.2.2021 | Decanted (20L snow) | 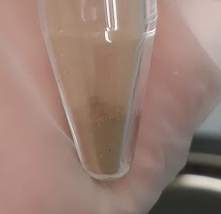 |  | X | X  0.12 |  |  |  |  |
| **101** | 61.50,  26.43  (Pertunmaa) | Not available | Filtered | 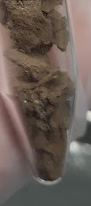 | X | X | X  2.6 | X | X | X  ** | X |
| **106** | 60.33,  25.09  (Vantaa) | Not available | Evaporated | 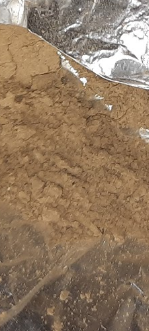 | X | X | X  1.1 | X |  | X  * | X |
| **115** | 60.40, 24.76  (Klaukkala) | 23.2.2021 | Evaporated (in sauna, 1 bucket of snow) | 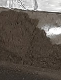 |  | X | X  0.48 |  |  |  | X |
| **206** | 61.57,  26.00  (Hartola) | 25.2.2021 | Filtered  (1 m^2^ surface snow) | 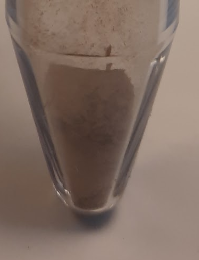 | X | X | X  0.24 | X |  | X  ** |  |
| **219** | 60.64,  26.14  (Lapin-  järvi) | 26.2.2021 | Evaporated  (3 dl snow) | 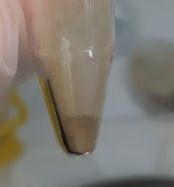 | X | X | X  0.05 | X |  |  |  |
| **273** | 60.17,  24.59  (Espoo) | 25.2.2021 | Filtered, (  5L snow) | 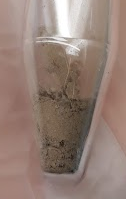 | X | X | X  0.20 | X |  | X  ** |  |
| **296** | 60.32,  25.02  (Vantaa) | 25.2.2021 | Evaporated | 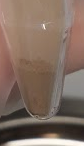 | X | X | X  0.20 | X |  | X  ** |  |
| **353** | 61.53,  24.22  (Kangasala) | 28.2.2021 | Filtered | 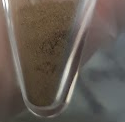 | X | X | X  0.20 | X |  |  |  |
| **485** | 62.42,  22.18  (Kauhajoki) | 24.2.2021 | Evaporated ( 15 kg dusty surface snow) | 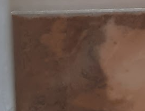 | X | X |  |  | X | X  ** |  |

**Supplementary Table S2.**  Colorimetry results in the L*a*b* system colour sphere, where *L** defines lightness from black at 0 and white at 100; *a** the green–red continuum, with negative values associated with green and positive values toward red; and *b** represents the blue–yellow continuum, with negative numbers toward blue and positive toward yellow.

| **Sample ID** | **L*(D65)** | **a*(D65)** | **b*(D65)** |
| --- | --- | --- | --- |
| **41** | 54.8 | 7.12 | 18.9 |
| **49** | 49.56 | 5.17 | 15.44 |
| **80** | 55.07 | 2.43 | 8.09 |
| **101** | 47.97 | 5.68 | 12.57 |
| **106** | 54.16 | 6.03 | 17.22 |
| **206** | 50.5 | 6.54 | 17.42 |
| **219** | 53.25 | 5.91 | 16.81 |
| **273** | 48.73 | 4.65 | 13.71 |
| **296** | 55.62 | 5.22 | 15.85 |
| **353** | 58.77 | 3.79 | 10.1 |

**Supplementary Table S3**. Concentrations of detected water soluble ions from citizen samples and the reference sample from Timbuktu.

|  | **Water soluble ions ng(ion)/mg(dust)** | | | | | | | |
| --- | --- | --- | --- | --- | --- | --- | --- | --- |
| **Sample** | **Na^+^** | **NH_4_^+^** | **K^+^** | **Mg_2_^+^** | **Ca_2_^+^** | **Cl** | **NO_3_** | **SO_4_** |
| **41** | 81.6 | 40.4 | 147.3 | 64.8 | 303.7 | 31.1 | 15.1 | 38.1 |
| **101** | 81.1 | 19.9 | 61.7 | 17.1 | 53.7 | 2.7 | 2.3 | 17.6 |
| **106** | 956.5 | 109.6 | 385.8 | 62.6 | 1043.8 | 564.7 | 99.5 | 545.3 |
| **115** | 49.6 | 31.8 | 45.5 | 16.5 | 70.2 | 54.1 | 1.2 | 7.4 |
| **474** | 56.7 | 256.3 | 166.4 | 61.1 | 190.9 | 6.8 | 7.2 | 55.9 |
| **Ref (Timbuktu)** | 9.3 | 23.0 | 53.6 | 9.6 | 59.4 | 8.0 | 12.7 | 19.2 |

**Supplementary Table S4**. Average optical depth at 550 nm, total atmospheric column, and deposition of aerosol species in SILAM over an area defined by latitudes [60,65] ^o^N and longitudes [21,28] ^o^E, roughly covering the southern half of Finland. The optical depth and total column have been computed as averages over the time span 00:00 to 15:00 UTC February 23. The average deposition has been computed for the five-day time span February 20 to February 24.

| **Species** | **Average optical depth** | **Average total column (mg/m^2^)** | **Average deposition (mg/m^2^)** |
| --- | --- | --- | --- |
| dust | 0.16 | 288 | 154 |
| organic carbon | 0.16 | 8.6 | 15 |
| nitrate | 0.05 | 43 | 1.1 |
| sulfate | 0.07 | 30 | 9.0 |
| sea salt | 0.01 | 0.6 | 2.2 |
| other species | 0.005 | 0.4 | 4.0 |
| all aerosol species | 0.45 | 370 | 194 |

**Supplementary Table S5.** Average physicochemical features for the source estimated by SILAM. These values cover only 57.5 % of the estimated source, as data were missing for the remaining part, which mainly (85 %) corresponded to arenosol as the soil type.

| sand fraction | 58.5 % weight |
| --- | --- |
| silt fraction | 26.0 % weight |
| clay fraction | 15.5 % weight |
| organic carbon | 0.62 % weight |
| pH in water | 7.7 (-log(H+)) |
| carbonates | 7.6 % weight |
| gypsum | 3.8 % weight |


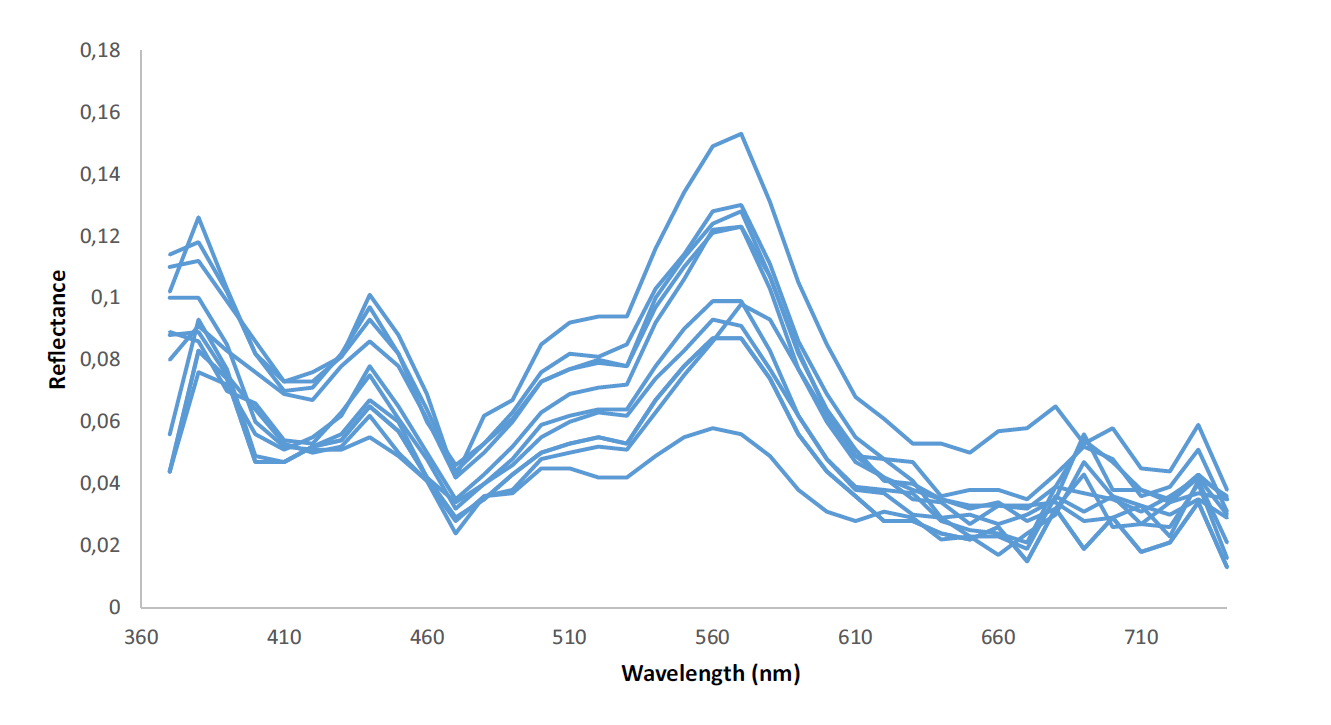


**Supplementary Figure S1.** The first derivative reflectances.


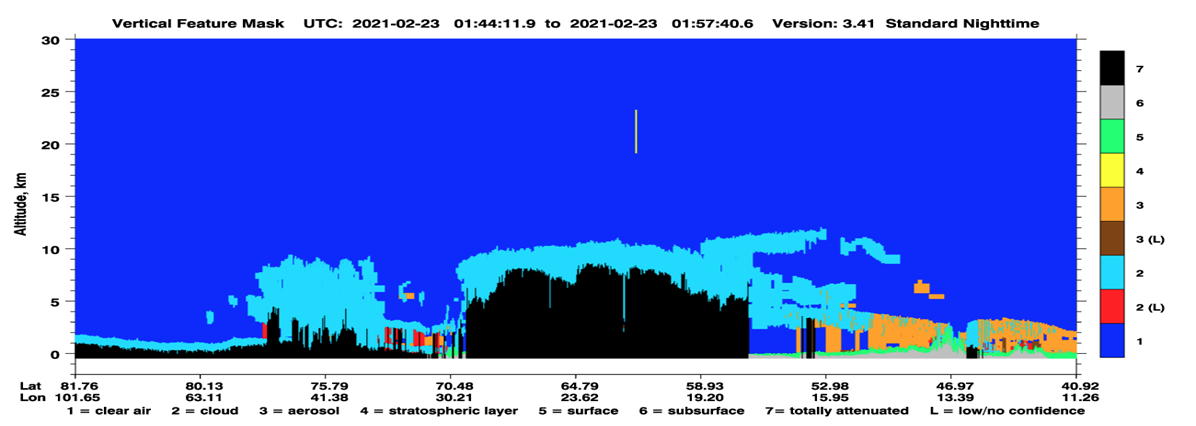


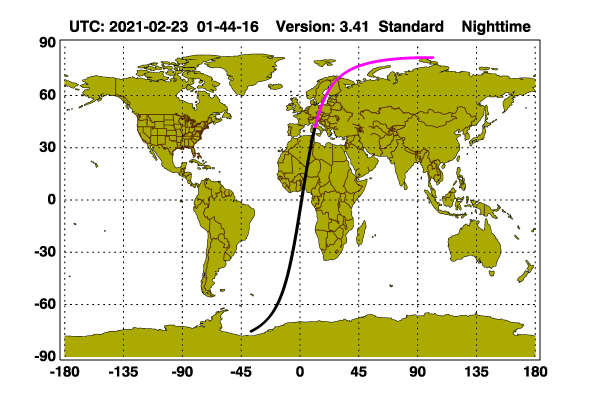


**Supplementary Figure S2.** Above: CALIPSO satellite data over Finland for 23 February 2021, which shows totally attenuated (black) and clouds (light blue) below 10 km at 60 - 70 ^o^N. Below: track of the satellite. Obtained from: https://www-calipso.larc.nasa.gov/products/lidar/browse_images/show_detail.php?s=production&v=V3-41&browse_date=2021-02-23&orbit_time=01-44-16&page=1&granule_name=CAL_LID_L1-ValStage1-V3-41.2021-02-23T01-44-16ZN.hdf. The figure was created using IDL. Version: 8.8.0. URL Link: https://www.nv5geospatialsoftware.com/Products/IDL/.


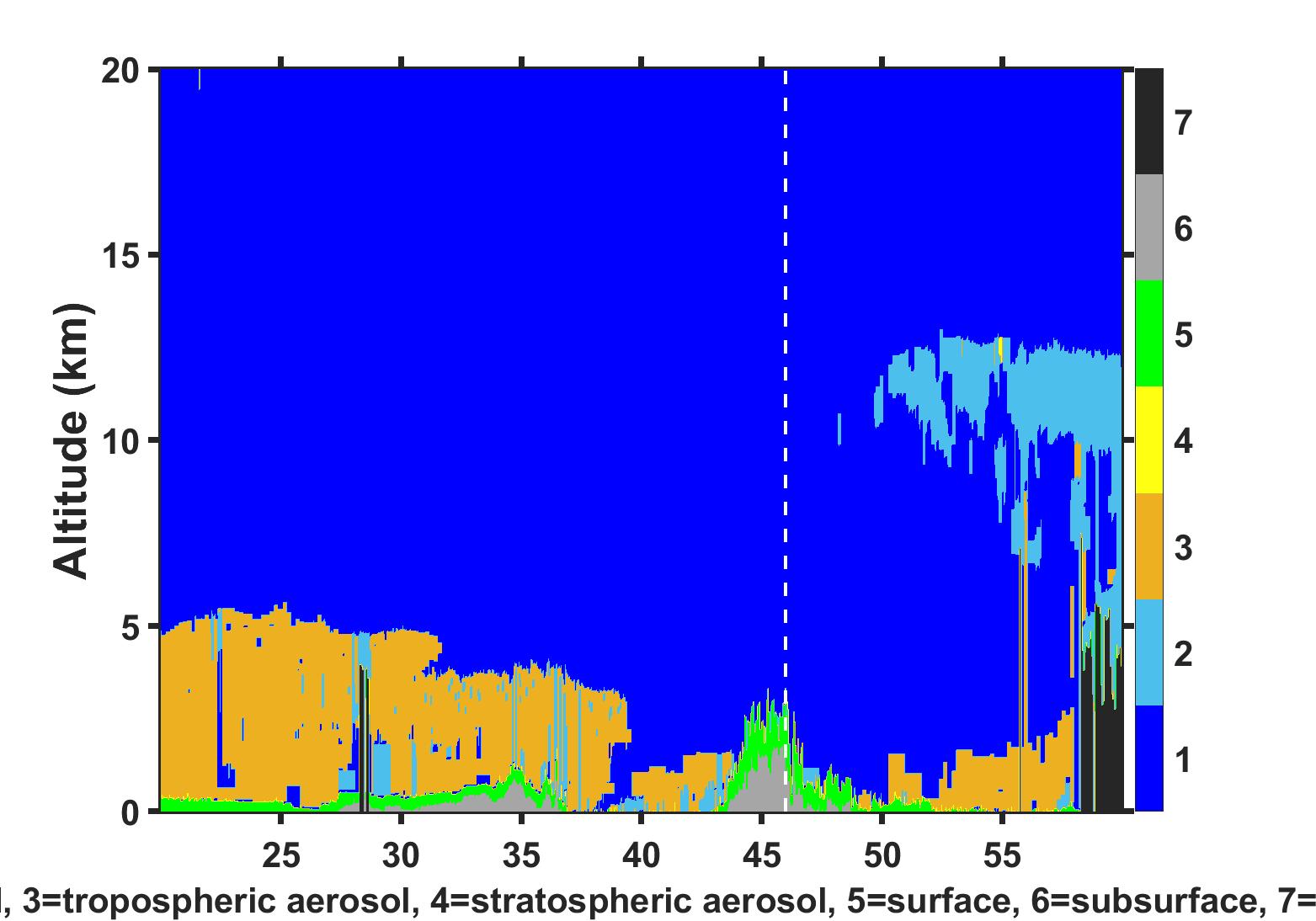


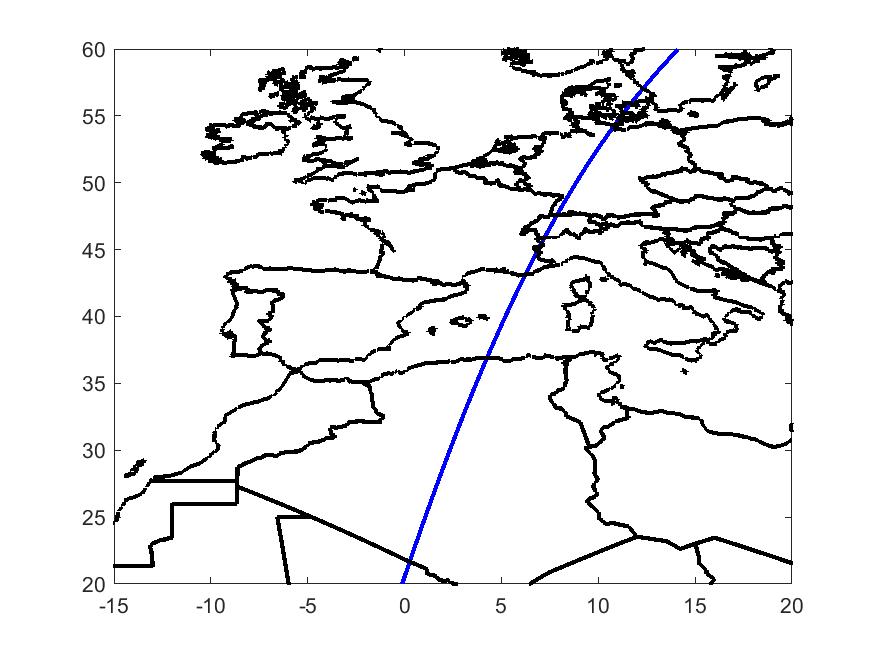


**Supplementary Figure S3**. Above: CALIPSO satellite data over the African source area for 21 February 2021, which show dust clouds below 5 km. Tropospheric aerosols are presented with orange color along the satellite track at 20 – 35 ^o^N. Below: Track of the satellite. Obtained from: https://www-calipso.larc.nasa.gov/products/lidar. The figure was created using IDL. Version: 8.8.0. URL Link: https://www.nv5geospatialsoftware.com/Products/IDL/.
