# Supplementary figures and images for: African dust transport and deposition modelling verified through a citizen science campaign in Finland
Source: Sci Rep. 2023 Dec 4;13:21379. doi: 10.1038/s41598-023-46321-7 (PMC10695925; doi:10.1038/s41598-023-46321-7)

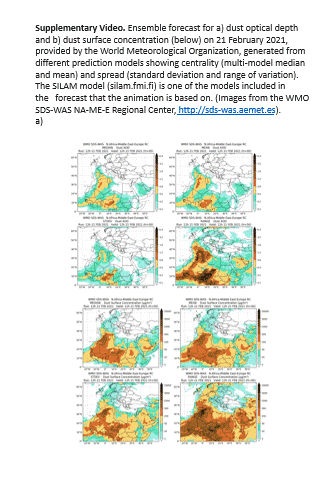

Supplement: Supplementary file 2 — Supplementary Video 1. [file 41598_2023_46321_MOESM2_ESM.gif]
